# Defining the three cell lineages of the human blastocyst by single-cell RNA-seq

**DOI:** 10.1242/dev.123547

**Published:** 2015-09-15

**Authors:** Paul Blakeley, Norah M. E. Fogarty, Ignacio del Valle, Sissy E. Wamaitha, Tim Xiaoming Hu, Kay Elder, Philip Snell, Leila Christie, Paul Robson, Kathy K. Niakan

**Affiliations:** 1 Human Embryology and Stem Cell Laboratory, The Francis Crick Institute, Mill Hill Laboratory, London NW7 1AA, UK; 2 Genome Institute of Singapore, A-STAR, Singapore 138672, Singapore; 3 MRC Functional Genomics Unit, Department of Physiology, Anatomy and Genetics, University of Oxford, Oxford OX1 3QX, UK; 4 Bourn Hall Clinic, Bourn, Cambridge CB23 2TN, UK; 5 The Jackson Laboratory for Genomic Medicine, Farmington, CT 06030, USA

**Keywords:** Human, Mouse, Epiblast, Trophectoderm, Embryonic stem cells, RNA-sequencing

## Abstract

Here, we provide fundamental insights into early human development by single-cell RNA-sequencing of human and mouse preimplantation embryos. We elucidate conserved transcriptional programs along with those that are human specific. Importantly, we validate our RNA-sequencing findings at the protein level, which further reveals differences in human and mouse embryo gene expression. For example, we identify several genes exclusively expressed in the human pluripotent epiblast, including the transcription factor *KLF17*. Key components of the TGF-β signalling pathway, including *NODAL*, *GDF3*, *TGFBR1/ALK5*, *LEFTY1*, *SMAD2*, *SMAD4* and *TDGF1*, are also enriched in the human epiblast. Intriguingly, inhibition of TGF-β signalling abrogates NANOG expression in human epiblast cells, consistent with a requirement for this pathway in pluripotency. Although the key trophectoderm factors *Id2*, *Elf5* and *Eomes* are exclusively localized to this lineage in the mouse, the human orthologues are either absent or expressed in alternative lineages. Importantly, we also identify genes with conserved expression dynamics, including *Foxa2/FOXA2*, which we show is restricted to the primitive endoderm in both human and mouse embryos. Comparison of the human epiblast to existing embryonic stem cells (hESCs) reveals conservation of pluripotency but also additional pathways more enriched in hESCs. Our analysis highlights significant differences in human preimplantation development compared with mouse and provides a molecular blueprint to understand human embryogenesis and its relationship to stem cells.

## Abstract (precis)

**Summary:** Single-cell RNA-sequencing of human and mouse embryos reveals conserved and human-specific transcriptional programmes as well as a functional requirement for TGFβ signalling in human embryos.

## INTRODUCTION

The morphology of the preimplantation human embryo is remarkably similar to the mouse embryo. After fertilization, both undergo mitotic cell divisions, compaction and cavitation to form a blastocyst comprised of a trophectoderm (TE) layer and an inner cell mass (ICM). Despite these similarities, there are a number of significant distinctions, such as the timing of cleavage divisions, blastocyst formation and implantation (Cockburn and Rossant, 2010; Niakan et al., 2012). Mouse embryos also undergo zygotic/embryo genome activation immediately after fertilization (Flach et al., 1982), whereas it remains unclear whether this occurs between the 4- and 8-cell stage or earlier in human embryos.

Three cell lineages comprise the blastocyst: pluripotent epiblast (EPI) cells that form the embryo proper, and extraembryonic TE cells and primitive endoderm (PE) cells that contribute to the placenta and yolk sac, respectively. The molecular mechanisms underlying the specification of these distinct lineages have been extensively studied in the mouse. In the mouse, the first cell fate decision, which segregates the ICM and TE, involves differential Hippo signalling at compaction (Nishioka et al., 2009). Differential FGF signalling at the blastocyst stage leads to the second cell fate decision, the segregation of the EPI and PE lineages within the ICM (Guo et al., 2010).

Comparatively little is known about mechanisms of lineage specification in human embryogenesis, although some gene expression patterns are shared with the mouse (Rossant, 2015). Like the mouse, human embryos express OCT4 in all cells until the blastocyst stage, when OCT4 is restricted to the EPI (Niakan and Eggan, 2013). Importantly, we previously found that the restriction of OCT4 expression to the EPI correlates with the optimal time for human embryonic stem cell (hESC) derivation, suggesting that further understanding of lineage specification will also have importance for stem cell biology (Chen et al., 2009). However, differences between these species in the expression of lineage-associated factors have also been noted. For example, in the mouse Cdx2 is expressed at the morula stage, whereas CDX2 expression follows cavitation in the human blastocyst (Niakan and Eggan, 2013).

Advances in single-cell RNA-sequencing (RNA-seq) transcriptomics approaches have provided significant insights into the transcriptional programs underlying human embryogenesis (Piras et al., 2014; Xue et al., 2013; Yan et al., 2013). Whereas previous studies have compared the transcriptomes of human and mouse preimplantation embryos (Piras et al., 2014; Xue et al., 2013), there is a limited focus on lineage specification. Additional studies used microarray analysis of whole embryos; however, cellular heterogeneity complicates the identification of cell-type specific gene expression (Madissoon et al., 2014; Xie et al., 2010; Zhang et al., 2009). Furthermore, few of these studies have validated their computational analyses with independent approaches. This is particularly important because of the known technical variability and stochastic expression in single-cell RNA measurements (Brennecke et al., 2013; Kim and Marioni, 2013) in addition to the threshold for expression having not yet been firmly established (Hebenstreit et al., 2011).

Here, we integrated our own human single-cell RNA-seq dataset with published human datasets and compared this with a published mouse single-cell dataset, allowing us to unravel novel temporal-, lineage- and species-specific factors. We developed a computational pipeline to cluster single cells into developmental stages based on their global gene expression profiles and showed that the major wave of embryo genome activation occurs between the 4- and 8-cell stage in human and between the zygote and late 2-cell stage in mouse. Our analysis revealed that temporal expression dynamics of key developmental regulators and their co-expressed genes are largely distinct in human versus mouse. Significantly, we resolved lineage-specific gene expression in humans, including expression of a number of key components of the TGF-β signalling pathway in the EPI. Treating human embryos with a potent TGF-β signalling inhibitor resulted in downregulation of NANOG, suggesting that this pathway is necessary to maintain the pluripotent EPI. Our analysis also uncovered factors with conserved expression in human and mouse embryos such as Foxa2/FOXA2, which was restricted to the PE. However, while we identified the transcription factor KLF17 as exclusively expressed in the human EPI, we found that the mouse EPI factors *Esrrb*, *Klf2* and *Bmp4* are absent from the human EPI. Moreover, a number of key mouse TE factors, including *Elf5* and *Eomes*, were absent in the human TE, and, conversely, human TE factors *CLDN10*, *PLAC8* and *TRIML1* were absent in the mouse. We found that although hESCs expressed many EPI-enriched genes, they also expressed genes that are absent in *in vivo* pluripotent cells. Altogether, we present a comprehensive comparison of human and mouse preimplantation development that reveals previously unappreciated differences in gene expression and highlights the importance of further analysing human preimplantation development rather than assuming equivalence to the mouse.

## RESULTS

### Comparative transcriptomics analysis throughout human and mouse preimplantation development reveals temporal differences in gene expression

To unravel similarities and differences between human and mouse embryogenesis, we compared their preimplantation transcriptomes using single-cell RNA-seq analysis. We used previously published human (Yan et al., 2013) and mouse (Deng et al., 2014) single-cell RNA-seq datasets as both include deep transcriptome profiling at comparable developmental stages, allowing comparative analysis of gene expression over time.

**Fig. 1.**
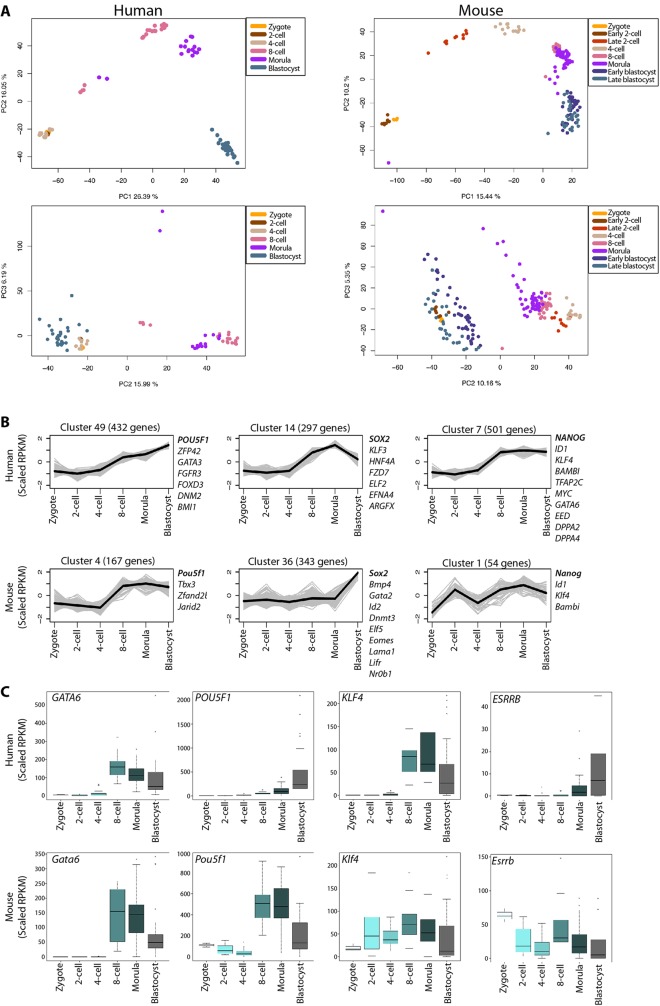
**Global gene expression dynamics in human and mouse preimplantation development.** (A) Principal component analysis of human (Yan et al., 2013) or mouse (Deng et al., 2014) single-cell RNA-seq transcriptomes. Each point represents a single cell and labelled according to developmental stage. Data were plotted along the first and second principal components and the second and third principal components. (B) K-means clusters showing selected genes co-expressed with *Pou5f1*/*POU5F1*, *Sox2*/*SOX2* or *Nanog*/*NANOG* in mouse or human pre-implantation embryos. Grey line corresponds to scaled RPKM values for genes and black line corresponds to median expression within the cluster. (C) Boxplots of RPKM values for selected genes showing the range of single-cell gene expression at each of the selected development stages. Boxes correspond to the first and third quartiles, horizontal line to the median, whiskers extend to 1.5 times the interquartile range and dots denote outliers.

To normalize for sequencing depth and transcript length, the reads per kilobase of exon model per million mapped reads (RPKM) method (Mortazavi et al., 2008) was applied to both datasets. For subsequent analysis of temporal changes in gene expression, genes were retained in both datasets if they were expressed in at least one sample, using an RPKM >5 threshold. This has been shown to capture putative functional mRNAs reliably (Hebenstreit et al., 2011) and is a more stringent threshold than RPKM ≥0.1 that was previously used (Yan et al., 2013). To investigate gene expression pattern variation between cells at a given stage and across time, we used principal components analysis (PCA) to identify single-cell samples with similar global gene expression patterns in human zygote, 2-cell, 4-cell, 8-cell, morula and late-blastocyst samples (Fig. 1A). As a comparison, we also performed a PCA of mouse zygote, early 2-cell, late 2-cell, 4-cell, 8-cell, morula, early-blastocyst and late-blastocyst samples. Whereas the plot of our *de novo* PCA of mouse samples closely resembles that previously reported (Deng et al., 2014), our PCA plot of the human samples is distinct from that by Yan et al., suggesting that this is due to different RPKM thresholds applied to the data.

The human and mouse PCA plots showed that the majority of single cells clustered according to their developmental stage. The compact cluster of the human zygote, 2-cell and 4-cell stage samples suggests that they are closer transcriptionally compared with later stages. Conversely in mouse, cells at the zygotic and early 2-cell stage clustered together, resulting in a clear distinction between late 2-cell and zygotic/early 2-cell stage. Therefore, the PCA suggests that the timing of embryo genome activation in human occurs between the 4- and 8-cell stages, consistent with previous experiments (Braude et al., 1988; Tesarík et al., 1987). Later in development, the human late-blastocyst samples clustered distinctly from the morula samples (Fig. 1A), suggesting that the human late blastocyst are more divergent in global gene expression.

To understand developmental gene expression dynamics further, we used k-means clustering to group genes with similar expression profiles in the human and mouse time-course data across development (Fig. 1B; supplementary material Figs S1, S2 and Tables S1, S2). We focused our analysis on genes with a fold change of more than two between any two developmental stages in each species. To determine the optimum number of k-means clusters, we used the Bayesian Information Criterion (BIC) score of the human data (supplementary material Fig. S3A), and therefore used 50 clusters in subsequent analyses.

The 50 k-means clusters of co-expressed genes were further grouped by hierarchical clustering (supplementary material Fig. S3B,C). Here, we observed two general patterns in both datasets. The first comprises genes that were highly expressed in the zygote and rapidly downregulated in subsequent stages, perhaps indicating maternal transcripts. The second comprises genes that were largely absent in the zygote and subsequently upregulated during or after zygotic/embryo genome activation. In mouse, clusters that were largely absent in the zygote were first upregulated at the 2-cell stage (*n*=10 clusters). By contrast, in human embryos, we first observed upregulation at the 4-cell stage (*n*=7 clusters), followed by the 8-cell stage (*n*=14 clusters). This is consistent with the onset of embryo genome activation at the 2-cell stage, and between the 4- and 8-cell stages in mouse and human, respectively.

To distinguish potentially conserved clusters of co-expressed genes, we selected the key pluripotency-associated factors *Pou5f1*/*POU5F1*, *Sox2*/*SOX2* and *Nanog*/*NANOG* and followed their temporal expression dynamics (Fig. 1B). *Pou5f1*/*POU5F1* (human cluster 49 and mouse cluster 4) shows an upregulation of expression from the 4-cell to the blastocyst stage. However, the genes co-expressed within these clusters were distinct between the species. For example, human *POU5F1* was co-expressed with the TE marker *GATA3*, the epigenetic regulator *BMI1*, and the pluripotency factors *ZFP42*/*REX1* and *FOXD3*, which were all absent in the corresponding mouse cluster. By contrast, *Pou5f1* was co-expressed with the epigenetic regulator *Jarid2* and the pluripotency factor *Tbx3*, which were absent in the corresponding human cluster. *SOX2* expression was upregulated from the 4-cell to the blastocyst stage in human (cluster 14) and was co-expressed with a number of genes, including *KLF3*, *FZD7*, *ELF2* and *HNF4A*. Mouse *Sox2* expression was highly upregulated at the blastocyst stage (cluster 36) and, interestingly, was co-expressed with a number of TE-associated genes, including *Gata2*, *Id2*, *Elf5* and *Eomes*. Whereas human *NANOG* expression was upregulated at the 4- to 8-cell stage (cluster 7), mouse *Nanog* expression was upregulated earlier between the zygotic and 2-cell stage (cluster 1). Intriguingly, *Id1*/*ID1*, *Klf4*/*KLF4* and *Bambi*/*BAMBI* were co-expressed with *Nanog*/*NANOG* in both species, suggesting that they belong to a conserved gene regulatory network. The *NANOG* cluster also contains a number of additional key developmental regulators such as the endoderm transcription factor *GATA6*, the epigenetic regulator *EED* and the pluripotency factors *DPPA2* and *DPPA4*. In all, largely distinct sets of genes co-expressed with these key pluripotency factors suggests alternative molecular programs operating between these species.

To resolve gene expression dynamics further, we generated boxplots of RPKM values across time (Fig. 1C). Importantly, the boxplots allow greater insight into variance of gene expression patterns. For some genes, such as *Gata6*/*GATA6*, we observed similar expression dynamics in both human and mouse embryos. However, the expression dynamics of most genes diverged between these species. For example, whereas *Klf4* was expressed from the earliest stages of mouse development and maintained thereafter, *KLF4* was first upregulated at the 8-cell stage in human. Similarly, *Esrrb* was expressed in mouse zygotes and maintained throughout preimplantation development, whereas *ESRRB* was expressed in human morulas and subsequently in blastocysts. *Pou5f1* transcripts were present in the mouse zygote and initially downregulated, followed by upregulation at the 8-cell stage. Interestingly, *POU5F1* was not present in the human embryo in appreciable levels until the 8-cell stage, suggesting that maternal transcripts present in human and mouse zygotes differ significantly. Altogether, this suggests that there are significant differences in gene expression dynamics across time in mouse and human embryos.

### Lineage-specific gene expression in human and mouse blastocysts

**Fig. 2.**
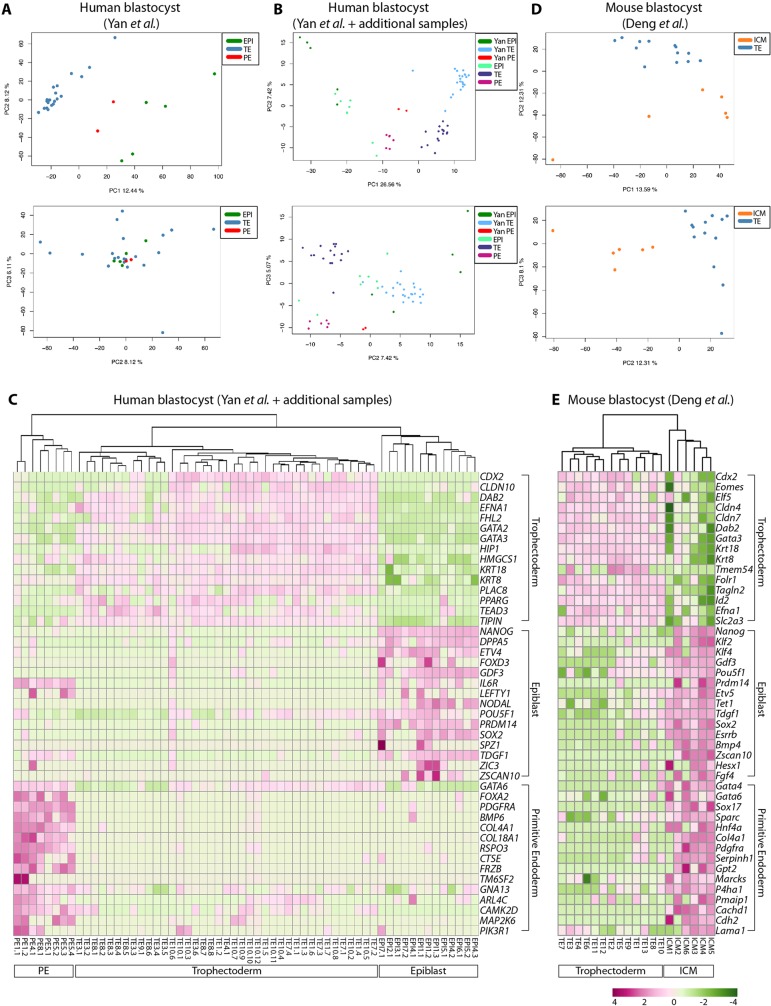
**Lineage-specific gene expression in human and mouse blastocysts.** (A,B,D) PCA at the late-blastocyst stage. Each point represents the gene expression profile of a single cell from blastocysts and labelled according to both lineage identity and experiment. Data were plotted along the first and second principal components and the second and third principal components. Data are from (A) Yan et al. (2013); (B) a combined dataset including our additional dataset together with data from Yan et al. (2013); (D) Deng et al. (2014). (C,E) Unsupervised hierarchical clustering of samples and heatmaps of differentially expressed genes. Normalized expression was plotted on a high-to-low scale (purple-white-green) and genes grouped according to lineage-associated expression. (C) A combined human late-blastocyst dataset including samples generated in our lab together with data from Yan et al. (2013). (E) Mouse late-blastocyst dataset from Deng et al. (2014).

To resolve lineage-specific gene expression in human blastocysts we initially used several unbiased approaches to distinguish cell type-specific gene expression in the late-blastocyst samples from Yan et al. We performed a PCA on the human late-blastocyst samples (Fig. 2A), which shows that projection onto the first two principal components was sufficient to group the human cells into two or three clusters. In parallel, we performed unsupervised hierarchical clustering, which reveals that the same samples cluster similarly into three groups (supplementary material Fig. S4A).

Given the limited number of single-cell EPI and PE samples analysed above, we aimed to increase the number of biological replicates to improve statistical power to detect differential gene expression. We performed RNA-seq of additional samples (*n*=30 cells; 7 embryos), followed by PCA combined with the time-course dataset from Yan et al. (supplementary material Fig. S4B). As expected, the additional samples clustered closer to the late-blastocyst stage samples from the Yan et al. dataset. A PCA of the blastocyst samples revealed that, while the additional EPI-assigned samples were intermingled with the Yan et al. EPI samples, the PE and TE samples were distinct on the PC2 and PC3 axes (Fig. 2B). These differences might be due to the inherent difficulty of matching developmental stages, differences in the single-cell cDNA synthesis and library preparation protocols or divergent genetic backgrounds. However, the samples do largely cluster into three lineage groups in the PCA as well as by an unsupervised hierarchical clustering (Fig. 2C).

To determine which lineage(s) these groups may correspond to, we generated a list of differentially expressed genes using NOISeq (Tarazona et al., 2011), a data-adaptive, non-parametric approach. This approach is well suited for single-cell RNA-seq analysis, as these data may not always conform to the same distributional assumptions as RNA-seq data from pooled cells (Kharchenko et al., 2014). NOISeq identified genes enriched in the presumptive EPI, including *NANOG*, *ETV4*, *PRDM14*, *FOXD3*, *POU5F1* and *SOX2* (Table 1; supplementary material Table S3). By contrast, the presumptive TE samples were enriched for genes including *GATA2*, *GATA3*, *CDX2* and *KRT18*, whereas the PE samples were enriched for *GATA4*, *GATA6*, *SOX17* and *COL4A1*. We also performed an independent test using DESeq (Anders and Huber, 2010), which fits a negative binomial model to the read count data, and observed considerable overlap of differentially expressed genes predicted by these two independent statistical methods (supplementary material Fig. S4C,D and Table S4). A heatmap of a subset of lineage-associated genes revealed that most of the human blastocyst samples exclusively expressed genes enriched in one of the lineages, suggesting that at this stage the cells were specified. As before, we found significant differences in the lineage assignments of several blastocyst samples when we compared our assignments with those of Yan et al. (supplementary material Fig. S4E). This further suggests that the RPKM threshold initially applied to determine expressed genes influences the conclusions drawn from subsequent analyses.

**Table 1.**
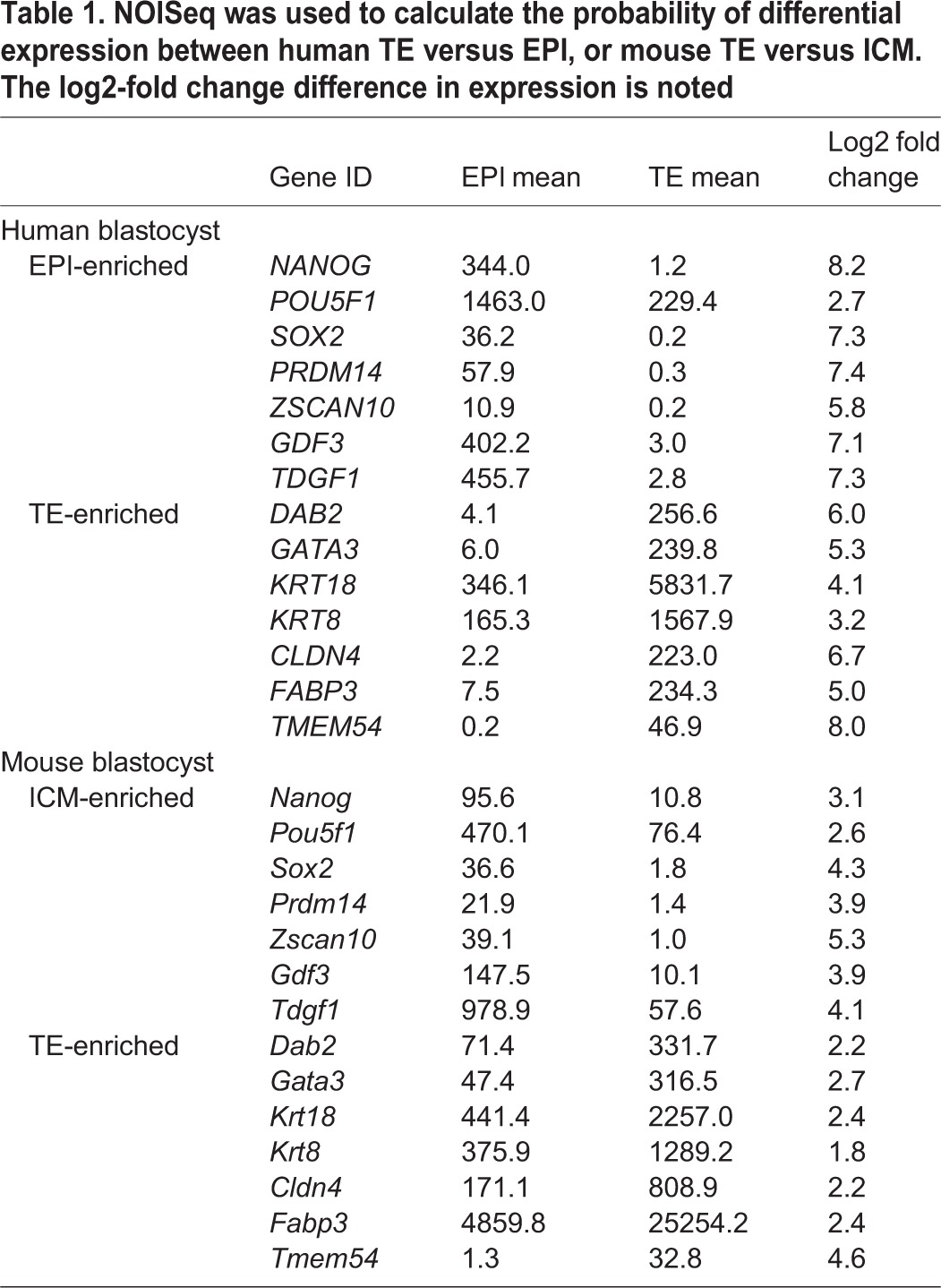
**NOISeq was used to calculate the probability of differential expression between human TE versus EPI, or mouse TE versus ICM. The log2-fold change difference in expression is noted**

**Fig. 3.**
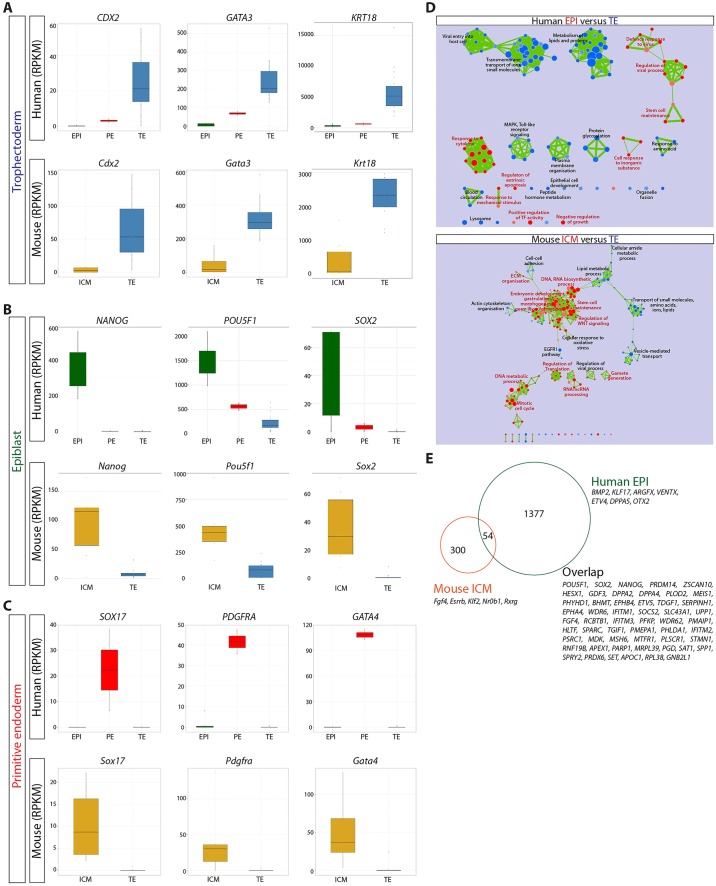
**Genes showing similar lineage-associated expression in human and mouse blastocysts.** (A) NOISeq was used to calculate the probability of differential expression between (A) human TE versus EPI, or mouse TE versus ICM. The log2-fold change (FC) difference in expression is noted. (A) Cytoscape enrichment map of GSEA results comparing human TE (blue) versus EPI (red), and mouse TE (blue) versus ICM (red) (*P*-value <0.01). (B-D) Boxplots of RPKM values for selected genes in human (Yan et al., 2013) or mouse (Deng et al., 2014) (B) TE; (C) EPI or (D) PE. The range of expression in human EPI (green), PE (red) or TE (blue) and in mouse ICM (orange) or TE (blue). Boxes correspond to the first and third quartiles, horizontal line to the median, whiskers extend to 1.5 times the interquartile range and dots were outliers. (E) Venn diagram of overlapping orthologous gene expression in human EPI and mouse ICM.

To distinguish pathways differentially enriched in either the EPI or TE we performed a comparative analysis of signalling pathways operating in these two lineages. Gene Set Enrichment Analysis (GSEA) (Subramanian et al., 2005) of human TE-enriched genes identified MAPK signalling, transmembrane transport of small molecules and metabolism of lipids and proteins among the most significantly enriched terms (Fig. 3A; supplementary material Fig. S5). By contrast, in the human EPI, GSEA showed that stem cell maintenance and TGF-β signalling were most significantly enriched. Altogether, this is consistent with appropriate lineage assignments for each human blastocyst cell as the pathways identified reflect expected biological characteristics of these lineages.

We next sought to determine lineage-specific expression in the mouse blastocyst samples. A PCA plotting both PC1 against PC2, and PC2 against PC3, distinguished a cluster of six cells from the remaining cells (Fig. 2D). NOISeq analysis identified 354 genes significantly enriched in these six ICM samples, including *Nanog*, *Pou5f1* and *Sox2*, compared with 143 genes in the remaining presumptive TE samples, which included *Gata3*, *Krt8* and *Krt18* (Table 1; supplementary material Table S5). Hierarchical clustering of these samples using the set of differentially expressed genes indicates a subset of EPI- and PE-associated genes that were simultaneously expressed in the six ICM-designated samples, including *Nanog*, *Esrrb*, *Sox2*, *Gata6*, *Sox17* and *Gata4* (Fig. 2E). This suggests that, while the mouse samples used in this study may have displayed morphological features of blastocyst formation, the ICM cells had not yet undergone lineage specification to EPI or PE. Interestingly, GSEA of mouse ICM-enriched genes revealed that stem cell maintenance, embryonic development and regulation of WNT signalling were among the most significantly enriched terms (Fig. 3A). By contrast, cell-cell adhesion, lipid metabolic process, transport of small molecules and EGFR1 pathway were significant terms for mouse TE-enriched genes.

Although the samples clustered into distinct lineages, within each group there was heterogeneity in levels of gene expression between individual cells. For example, in the human EPI cells, which expressed consistently high *NANOG* and *DPPA5*, we see variable expression of *POU5F1* and *SOX2* (Fig. 2B). Similarly, the variability in *Pou5f1* expression in the mouse ICM was also captured in the single-cell transcriptomics analysis. However, *PDGFRA*, *COL4A1* and *RSPO3* were consistently expressed in the human PE, suggesting that these are informative markers of this lineage. The observed heterogeneity in gene expression between single cells, even for key transcriptional regulators, highlights the need for including multiple replicate samples when studying lineage-specific gene expression.

### Comparison of lineage-specific gene expression in human and mouse blastocysts

Next, we investigated genes that were conserved in their lineage-specific expression. Key TE-associated genes *Cdx2*/*CDX2*, *Gata3*/*GATA3* and *Krt18*/*KRT18* were more highly expressed in this lineage in both human and mouse (Fig. 3B). Comparative analysis between human EPI and the mouse ICM revealed 54 orthologous genes enriched in these lineages relative to their respective TE (Fig. 3C,E), including core pluripotency factors *Nanog*/*NANOG*, *Pou5f1*/*POU5F1* and *Sox2*/*SOX2*. Moreover, a number of additional genes thought to function in regulating pluripotency were also conserved, including *Prdm14*/*PRDM14*, *Klf4*/*KLF4*, *Dppa4*/*DPPA4*, *Hesx1*/*HESX1*, *Dppa2*/*DPPA2, Tdgf1*/*TDGF1* and *Gdf3*/*GDF3* (Fig. 3E). This suggests that there are additional genes within the overlapping set that have a conserved role in the pluripotent EPI but the function of which has not yet been explored. The PE-associated genes *Sox17*/*SOX17*, *Pdgfra*/*PDGFRA* and *Gata4*/*GATA4* also showed conserved enrichment in the human PE and the mouse ICM (Fig. 3D).

**Fig. 4.**
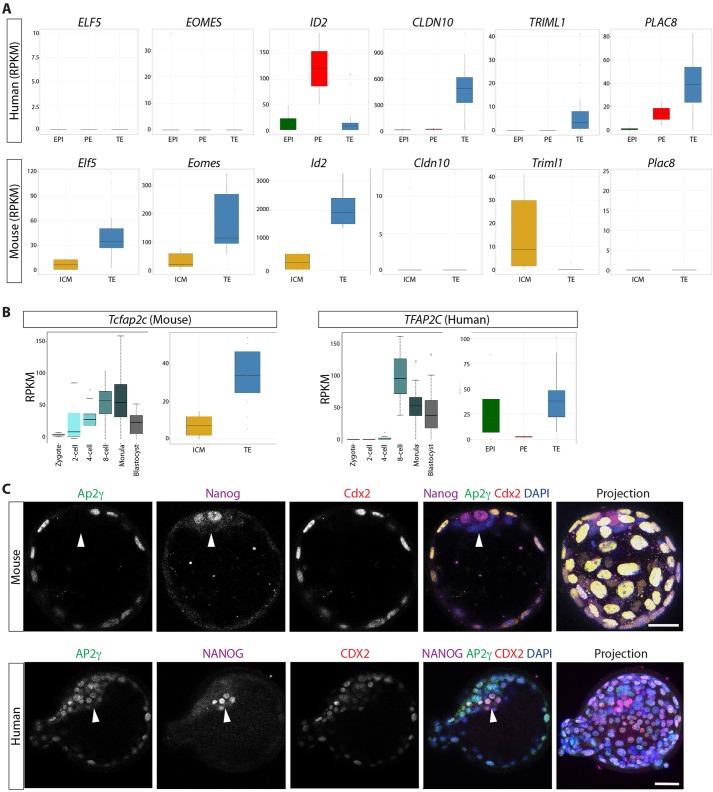
**Differences in TE-associated gene expression in human versus mouse blastocysts.** (A) Boxplots of RPKM values for selected genes. The range of expression in human EPI (green), PE (red) or TE (blue) and in mouse ICM (orange) or TE (blue). Boxes correspond to the first and third quartiles, horizontal line to the median, whiskers extend to 1.5 times the interquartile range and dots were outliers. (B) Boxplots of RPKM values for *Tcfap2c*/*TFAP2C* in human or mouse late-blastocysts and at each of the selected development stages. (C) Immunofluorescence analysis of human or mouse blastocysts for Ap2γ/AP2γ (green), Nanog/NANOG (purple), Cdx2/CDX2 (red) or DAPI (blue) with merged and projection images. Arrowheads indicate the location of the inner cell mass. Scale bars: 25 µm.

Although we identified a number of genes with conserved expression, we also observe important differences. Significantly, although *Elf5*, *Eomes* and *Id2* were highly enriched in the mouse TE, *ELF5* and *EOMES* were completely absent from any of the lineages in human, and *ID2* was most abundantly expressed in the PE and absent from most TE cells (Fig. 4A). We also observed genes highly enriched in the human TE, which were not expressed in mouse TE, including *Cldn10*/*CLDN10*, *Triml1*/*TRIML1* and *Plac8*/*PLAC8*, demonstrating key differences in TE gene expression between human and mouse. Furthermore, we find that *Tcfap2c*, a key transcriptional regulator in the mouse TE, had a different expression pattern in the human (Fig. 4B). Our RNA-seq analysis detected *Tcfap2c* transcripts in the mouse zygote, with levels remaining high as development proceeds. By contrast, abundant expression of the *Tcfap2c* orthologue *TFAP2C* was first detected at the 8-cell stage in human embryos. Lineage-specific analysis showed that, as expected, *Tcfap2c* was enriched in the mouse TE. By contrast, in the human blastocyst *TFAP2C* was expressed at similar levels in both the TE and EPI. Immunofluorescence analysis confirmed that Ap2γ, the protein product of *Tcfap2c*, was specifically localized to Cdx2^+^ TE cells in mouse and absent from Nanog^+^ cells within the ICM (Fig. 4C; supplementary material Fig. S5). By contrast, AP2γ was detected in both CDX2^+^ TE cells and NANOG^+^ EPI cells in human blastocysts (Fig. 4C; supplementary material Fig. S6).

**Fig. 5.**
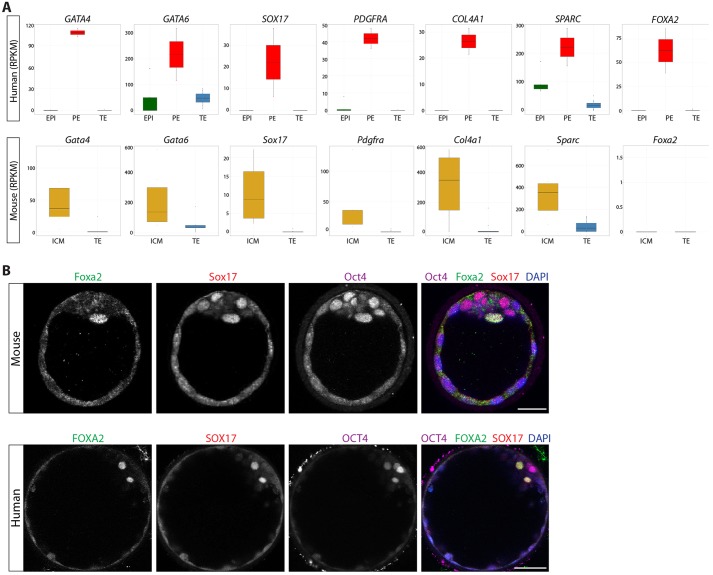
**Similarities in the expression of PE-associated genes in human and mouse blastocysts.** (A) Boxplots of RPKM values for selected genes. The range of expression in human EPI (green), PE (red) or TE (blue) and in mouse ICM (orange) or TE (blue). Boxes correspond to the first and third quartiles, horizontal line to the median, whiskers extend to 1.5 times the interquartile range and dots were outliers. (B) Immunofluorescence analysis of human or mouse blastocysts for Foxa2/FOXA2 (green), Sox17/SOX17 (red), Oct4/OCT4 (purple) or DAPI (blue) with merged images. Scale bars: 25 µm.

We investigated the conservation of PE-associated genes in human and mouse. *Gata4*, *Gata6*, *Sox17*, *Pdgfra*, *Col4a1* and *Sparc* are known to be associated with the mouse PE or its derivatives, with many functionally required for this lineage (Schrode et al., 2013). As expected, we observe abundant expression of these genes in the mouse ICM, and their human orthologues were also more highly expressed in the human PE (Fig. 5A). Hierarchical clustering revealed that human PE cells expressed *FOXA2* (Fig. 2B), a gene typically associated with later endoderm development (Ang and Rossant, 1994; Ang et al., 1993; Monaghan et al., 1993; Sasaki and Hogan, 1993). The boxplots confirmed lineage-specific expression of *FOXA2* in the human PE, whereas we failed to detect *Foxa2* expression in the mouse samples analysed (Fig. 5A). Significantly, immunofluorescence analysis further confirmed that FOXA2 protein was specifically localized to the human PE where it was co-expressed with SOX17, indicating that it is a novel marker of this lineage (Fig. 5B; supplementary material Fig. S7A). We observe co-localisation of Foxa2 with a subset of Sox17-expressing cells in the mouse late-blastocyst (supplementary material Fig. S7B) but failed to detect Foxa2 in earlier-stage embryos (data not shown), suggesting that Foxa2 is a marker of the mouse late PE. This might explain the absence of *Foxa2* expression in the mouse transcriptome dataset, which appears to have captured expression prior to the late-blastocyst stage, consistent with the co-expression of EPI- and PE-associated transcripts detected in these samples (Fig. 2E).

**Fig. 6.**
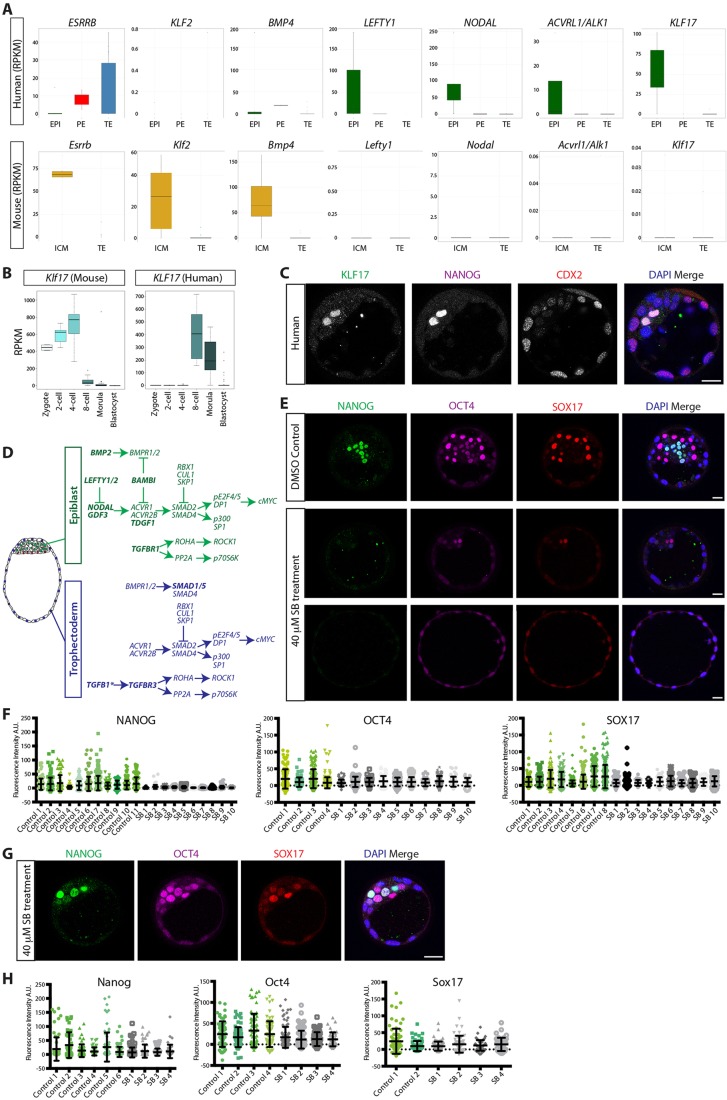
**Differences in the expression of EPI-associated genes in human versus mouse blastocysts.** (A) Boxplots of RPKM values for selected genes. The range of expression in human EPI (green), PE (red) or TE (blue) and in mouse ICM (orange) or TE (blue). Boxes correspond to the first and third quartiles, horizontal line to the median, whiskers extend to 1.5 times the interquartile range and dots were outliers. (B) Boxplots of RPKM values for *Klf17*/*KLF17* in human or mouse at each of the selected development stages. (C) Immunofluorescence analysis of human blastocysts for KLF17 (green), NANOG (purple), CDX2 (red) or DAPI (blue) with merged image. Scale bars: 25 µm. (D) Summary of TGF-β signalling components expressed at an RPKM value >5 in human EPI or TE. Bold denotes differentially expressed genes. *Indicates genes the expression of which falls just below the RPKM threshold. (E) Immunofluorescence analysis of SB-431542-treated or DMSO control human embryos for NANOG (green), OCT4 (purple), SOX17 (red) or DAPI (blue) with merged images. Scale bars: 25 µm. (F) Fluorescence intensity of NANOG, OCT4 or SOX17 in individual cells in each control or SB-431542 (SB)-treated embryo. (G) Immunofluorescence analysis of SB-431542-treated mouse embryos for Nanog (green), Oct4 (purple), Sox17 (red) or DAPI (blue) with merged image. Scale bar: 25 µm. (H) Fluorescence intensity of Nanog, Oct4 or Sox17 in individual cells in each control or SB-431542 (SB)-treated embryo.

Several genes were differentially expressed between the human EPI and mouse ICM (Fig. 3E). Importantly, while the known mouse pluripotency-associated factors *Esrrb*, *Klf2* and *Bmp4* (Nichols and Smith, 2012) were highly enriched in the mouse ICM, *KLF2* was absent from the human blastocysts, and *ESRRB* and *BMP4* were largely restricted to PE and/or TE cells (Fig. 6A). Conversely, we observed genes that were highly enriched in the human EPI, such as *LEFTY1*, *NODAL* and *ACVRL1/ALK1*, which were not expressed in mouse ICM at this stage (Fig. 6A). However, components of TGF-β signalling pathway, including Activins, *Nodal* and *Lefty1*, are expressed in mouse preimplantation embryos as early as E3.5 (Albano et al., 1993; Paria et al., 1992; Takaoka et al., 2011; Varlet et al., 1997). Given the absence of these factors from the dataset, this further suggests that the mouse ICM samples used reflect an earlier stage of blastocyst development.

Significantly, we also identified several transcription factors that were uniquely enriched in human EPI cells, including *KLF17*, which was initially expressed at the 8-cell stage in human embryos and highly enriched in human EPI cells (Fig. 6B). Despite expression in earlier stages of development, *Klf17* was absent in mouse blastocysts (Fig. 6B). By immunofluorescence analysis, we confirmed that KLF17 expression co-localised with NANOG within the EPI cells of human embryos (Fig. 6C) but was undetectable in mouse late-blastocysts (data not shown). Altogether, the single-cell RNA-seq analysis and subsequent validation allowed the confirmation of lineage-associated gene expression, thereby revealing fundamental differences in the expression of factors in human and mouse blastocysts.

### TGF-β signalling is necessary for the maintenance of NANOG in human pluripotent EPI cells

We observed robust expression of multiple components of the TGF-β signalling pathway in the human blastocyst, including *SMAD2* and *SMAD4* and receptors *ACVR1*, *ACVR2B*, *BMPR1* and *BMPR2*. Interestingly, there were differences between the EPI and TE lineages (Fig. 6D). Receptors *TDGF1* and *TGFBR1*, and ligands *NODAL*, *GDF3* and *BMP2*, were enriched in the EPI, whereas the TE showed enriched expression for *TGFB1* and the negative regulator *TGFBR3*. The expression of negative regulators *LEFTY1*, *LEFTY2* and *BAMBI* in the EPI indicated a feedback loop regulating this pathway. Moreover, in addition to *SMAD2* and *SMAD4*, the TE also expressed *SMAD1* and *SMAD5*, further suggesting that TGF-β signalling differentially regulates these lineages.

Components of the TGF-β signalling pathway are also expressed in hESCs (Besser, 2004; James et al., 2005; Levine and Brivanlou, 2006; Sato et al., 2003; Vallier et al., 2009). TGF-β signalling contributes to the maintenance of hESCs by regulating pluripotency gene expression (Bertero et al., 2015; Brown et al., 2011; James et al., 2005; Vallier et al., 2005, 2004; Xu et al., 2008). Given this role in hESCs, we sought to determine whether this pathway was functionally required for the EPI. We treated human embryos from E3 to E5 with the selective Activin receptor inhibitor SB-431542 at a concentration of 40 µM, which has been shown to block TGF-β signalling in mouse embryos effectively without toxicity (Granier et al., 2011), and which, as we confirmed, downregulated NANOG expression in hESCs (supplementary material Fig. S8). We performed immunofluorescence analysis of NANOG and OCT4 expression in blastocysts at E6-E7. Significantly, most human embryos lacked detectable NANOG expression in the presence of the inhibitor (Fig. 6E,F). Moreover, SOX17 expression was also undetectable in the majority of treated embryos. Whereas OCT4 expression was observed, there were fewer embryos with OCT4-high expressing cells compared with controls. Altogether, this suggests that TGF-β signalling is required to maintain key pluripotency marker expression in human EPI cells and a PE marker *in vivo*.

A Smad2/3-dependent autoregulatory loop is present in mouse preimplantation embryos, indicating a role for TGF-β signalling (Granier et al., 2011; Papanayotou and Collignon, 2014). While EPI formation is initiated, by E5.0 EPI and extraembryonic endoderm genes are mis-expressed in both TGF-β signalling-mutant and SB-431542-treated embryos, and further development is compromised (Brennan et al., 2001; Camus et al., 2006; Mesnard et al., 2006; Robertson et al., 2003; Waldrip et al., 1998). Treatment of mouse embryos from the 8-cell to blastocyst stage with SB-431542 does not affect the number of Oct4- or Gata4-expressing cells prior to implantation at E4.5 (Granier et al., 2011). However, as Nanog expression had not yet been examined in SB-431542-treated mouse embryos, we sought to determine whether there might be an effect on its expression. In contrast to the human, we found no effect on Nanog, Oct4 or Sox17 expression in treated mouse embryos, which robustly expressed all three markers (Fig. 6G,H), similar to controls. This further suggests that, while TGF-β signalling is active prior to implantation in mouse embryos, it is not required to initiate or maintain the expression of these EPI or PE markers.

### Defining human ground state pluripotency

Existing hESCs are thought to represent a later stage of development than their mESC counterparts, despite both being derived from preimplantation blastocysts. Indeed, hESCs share several characteristics with postimplantation-derived mouse epiblast stem cells (EpiSCs), including morphological similarities, LIF-independent growth and a reliance on FGF and Activin/Nodal signalling (Brons et al., 2007; Tesar et al., 2007). Addition of Mek and Gsk3b inhibitors together with LIF (2i+LIF) allows mESCs to be propagated in defined medium thought to represent a ‘ground state’ of pluripotency that is more similar to mouse preimplantation EPI cells, as compared with classical serum and LIF mESCs (Boroviak et al., 2014; Ying et al., 2008). Recent attempts to derive ground state hESCs have utilised combinations of ectopic transgene expression, growth factors and inhibitors to modulate signalling pathways (Chan et al., 2013; Gafni et al., 2013; Takashima et al., 2014; Theunissen et al., 2014). Mek and Gsk3b inhibitors are often included, although 2i+LIF alone is unable to support the self-renewal of hESCs (Hanna et al., 2010). However, the benchmark against which these cells are assessed relies heavily on conclusions drawn from mouse ground state pluripotency, which our analysis suggests not to be equivalent to the human EPI.

**Fig. 7.**
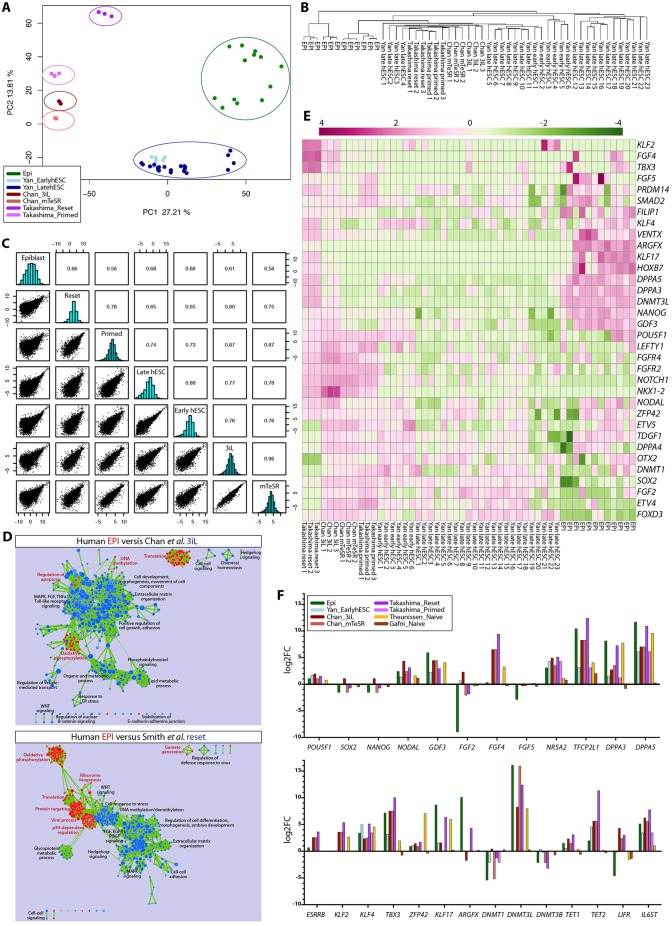
**Defining human ground state pluripotency.** (A) PCA of human EPI and hESCs grown in distinct culture conditions. Each point represents the gene expression profile of a single cell from the human EPI, single cell from Yan et al. late or early hESCs, clumps of hESCs from either Chan et al. (3iL or mTeSR) or Takashima et al. (reset or primed). (B) Unsupervised hierarchical clustering of global gene expression of human EPI or hESCs. (C) Pearson correlation coefficient between each pair of conditions indicated. (D) Cytoscape enrichment map of GSEA results comparing human EPI (red) versus 3iL or reset hESCs (blue) (*P*-value <0.01). (E) Heatmaps of selected differentially expressed genes in human EPI and hESCs. Expression levels were plotted on a high-to-low scale (purple-white-green). (F) The log2 fold change for selected genes in each condition relative to the expression of hESCs maintained on MEFs.

We compared the human EPI to various hESCs using NOISeq to determine the extent to which their gene expression profiles represented the EPI programme (Chan et al., 2013; Takashima et al., 2014; Yan et al., 2014). PCA of differentially expressed genes revealed that samples largely clustered according to experimental condition and cell type (Fig. 7A). We performed unsupervised hierarchical clustering of global gene expression, which again showed that the EPI samples clustered distinctly from hESCs (Fig. 7B). Calculating the Pearson correlation coefficient between each pair of conditions indicated that the hESCs all generally remained distinct from the EPI, with correlation values ranging from 0.58 to 0.68 (Fig. 7C).

We performed GSEA to identify differentially enriched pathways between the EPI and each hESC condition (Fig. 7D). We found that the EPI was enriched for oxidative phosphorylation signalling (Fig. 7D; supplementary material Fig. S5), possibly reflecting the switch to glycolytic metabolism following stem cell derivation in oxygen-rich conditions (Zhang et al., 2011). hESCs were enriched for regulation of cell proliferation (Fig. 7D; supplementary material Fig. S5). This suggested that a number of the distinctions were linked to intrinsic properties required to maintain the stem cell state. hESCs were also enriched for FGF, MAPK and Wnt signalling pathways (Fig. 7D; supplementary material Fig. S5). Significantly, both the EPI and hESCs expressed a number of key pluripotency genes, including *NANOG*, *NODAL* and *PRDM14* (Fig. 7E). Intriguingly, the Chan et al. 3iL and Takashima et al. reset hESCs cultured in alternative conditions upregulated EPI-enriched genes that were not appreciably expressed in conventional hESCs, including *DPPA3*, *DPPA5* and *DNMT3L* (Fig. 7E,F), suggesting that these conditions have indeed promoted an EPI-like gene expression profile.

We next integrated microarray analyses of additional alternative hESCs (Gafni et al., 2013; Theunissen et al., 2014) with the RNA-seq datasets by normalising the expression of all samples to conventional hESC derivation conditions (MEFs plus exogenous FGF). As expected, *NANOG*, *POU5F1* and *SOX2* expression was similar in both the EPI and hESCs (Fig. 7F), and *NODAL* and *GDF3* were also upregulated, reflecting the requirement for TGF-β signalling in maintaining NANOG expression in the human EPI (Fig. 6E). Furthermore, EPI-associated genes, including *NR5A2*, *TFCP2L1*, *DPPA3* and *DPPA5*, were expressed in several of the hESCs*.* However, we found inappropriate upregulation of additional signalling factors *FGF2* and *FGF4* and the LIF receptor *LIFR* in hESCs, although, curiously, the LIF co-receptor *IL6ST* (*GP130*) was also enriched in the EPI (Fig. 7F). Although some factors associated with the mouse ground state, such as *KLF4*, *TBX3* and *DNTM3L*, were upregulated in both the EPI and hESCs, others, including *ESRRB* and *KLF2*, were not appreciably expressed in the human EPI. Interestingly, the novel EPI-specific transcription factor that we identified as *KLF17* was upregulated specifically in the Takashima et al. reset and Theunissen et al. naïve cells (Fig. 7F). Altogether, this suggests that some of the alternative hESC culture conditions do indeed promote a programme closer to that of the human EPI, but extraneous signalling pathway activation might explain why these cells remain distinct. It would be interesting to determine how best these pathways could be modulated to fully reflect the human EPI.

## DISCUSSION

Our robust computational analyses of single-cell RNA-seq datasets revealed a number of novel temporal-, lineage- and species-specific factors in human and mouse embryos. Our findings have significance for stem cell biology, as the gene networks and signalling pathways regulating human pluripotency during development have yet to be elucidated and this work provides a molecular blueprint to uncover these mechanisms.

Using multiple independent data-mining approaches, our analysis suggests a single wave of genome activation between the 4-cell and 8-cell stage in human embryos, thereby supporting conclusions from uracil radiolabelling and alpha-amanitin transcriptional inhibition experiments (Braude et al., 1988; Tesarík et al., 1987). This is in contrast to findings that suggest a minor wave of genome activation and transcript upregulation before the 4-cell stage in human (Dobson et al., 2004; Xue et al., 2013). The apparent early detection of transcripts could be due to a subset of preferentially stable transcripts, or, alternatively, the delayed polyadenylation of maternal mRNAs (Aanes et al., 2011). To resolve this discrepancy, it might be possible to combine new advances in single-cell transcriptomics together with techniques to enrich for nascent RNA production (Jao and Salic, 2008) to distinguish embryonically transcribed mRNAs.

In the mouse, *Id2* and *Cdx2* are among the earliest transcription factors expressed in TE cells, followed by the expression of *Eomes* and *Elf5* (Guo et al., 2010; Ng et al., 2008; Russ et al., 2000; Strumpf et al., 2005). Our surprising discovery that most of these factors are absent in the human TE suggests that there are fundamental species differences in TE specification, consistent with the temporal differences in CDX2 expression we reported previously (Niakan and Eggan, 2013). In the mouse, *Tcfap2c* is required for the maintenance of the TE lineage, and induced expression of *Tcfap2c* in mESCs is sufficient to derive mouse trophoblast stem cells (Auman et al., 2002; Kuckenberg et al., 2010; Werling and Schorle, 2002). In the human placenta, *TFAP2C* is expressed in all trophoblast lineages (Biadasiewicz et al., 2011). Our finding that *TFAP2C* is more broadly expressed in the human blastocyst is a significant cautionary note against using this gene to assess TE identity. We propose *CLDN10*, *PLAC8* and *TRIML1* along with others identified in our analysis as candidates to distinguish TE cells more appropriately.

We have identified human-specific EPI-enriched genes, such as *KLF17*. As alternative members of the KLF family are involved in pluripotency, it would be interesting to investigate whether *KLF17* might replace known reprogramming factors, such as *Klf4*, and to determine its function in alternative hESCs. Furthermore, additional gene networks were enriched in both conventional and alternative hESCs compared with the human EPI, including the FGF and Wnt signalling pathway. Given differences in the signalling environment in the human EPI compared with hESCs that were noted previously (Kuijk et al., 2012; Kunath et al., 2014; Roode et al., 2012) and highlighted in this study, it will be intriguing to investigate the possibility of a distinct human pluripotent state further.

Recent work has suggested that distinct genetic programs and signalling pathways involved in lineage specification exist in human and mouse blastocysts, for example the differential requirement for FGF signalling in EPI and PE lineage specification (Kuijk et al., 2012; Kunath et al., 2014; Lanner and Rossant, 2010; Roode et al., 2012). Significantly, we found that several key TGF-β signalling pathway components were highly enriched and differentially expressed in the human EPI and TE, and that inhibiting this pathway led to downregulation of NANOG expression in human but not mouse EPI cells. It was previously suggested that TGF-β signalling inhibition increases EPI proliferation and enhances the outgrowth of cells during hESC derivation (Van der Jeught et al., 2014). The discrepancy with our results might be due to the fourfold lower concentration of SB-431542 used in the previous study as well as presence of mouse embryonic fibroblasts, known to secrete factors promoting TGF-β signalling, during the hESC derivations described, suggesting that this pathway has not been completely abolished. Altogether, this suggests that TGF-β signalling is required for the development of the pluripotent EPI in human blastocysts and further supports the requirement of this signalling pathway in pluripotent hESCs. It would therefore be interesting to determine whether stimulating TGF-β signalling in the absence of FGFs during hESC derivation might better recapitulate the embryo signalling environment. Finally, additional alternative signalling pathways might be required for the development of the human EPI and subsequent stem cell derivation. Our dataset provides a resource to discover these developmental cues.

## MATERIALS AND METHODS

### Human embryo culture and manipulation

Human embryos were donated to the research project by informed consent under the UK Human Fertilisation and Authority Licence number R0162. Embryos were thawed according to recommendations from Bourn Hall Clinic, the *in vitro* fertilization clinic coordinating donations. Single cells were isolated with the assistance of a Saturn 5 laser (Research Instruments). Further details of the protocols can be found in supplementary material Methods.

### Immunofluorescence analysis

Samples were fixed in 4% paraformaldehyde at 4°C for 1 h and immunofluorescently analysed as described previously (Niakan and Eggan, 2013). The primary antibodies (all at 1:500 dilution) used include: anti-Oct4 (sc-5279, sc-8628 or sc-9081, Santa Cruz Biotech), anti-Nanog (AF1997 R&D, REC-RCAB0001P 2B Scientific, or ab21624, Abcam), anti-Cdx2 (MU392A-UC, Biogenex), anti-Klf17 (HPA024629, Atlas), anti-Ap2γ (AF5059, R&D), anti-Sox17 (AF1924, R&D) and anti-Foxa2 (3143, Cell Signaling). Embryos were imaged on a Leica SP5 inverted confocal microscope (Leica Microsystems).

### cDNA synthesis, shearing and library preparation

RNA was extracted from single cells and processed for cDNA synthesis using the SMARTer Ultra Low RNA Kit for Illumina Sequencing-HV (Clontech Laboratories). Libraries were prepared using Clontech Low Input Library Prep Kit according to the manufacturer's instructions. An extended protocol can be found in the supplementary material Methods.

### Data acquisition and processing

Human and mouse single-cell RNA-seq data normalised using the RPKM method were taken from two previous publications (Deng et al., 2014; Yan et al., 2013) and integrated with our own blastocyst sequencing data. We filtered these datasets, retaining only genes having RPKM >5 in at least one sample. Extended methods can be found in supplementary material Methods.

Data have been deposited into Gene Expression Omnibus (GEO66507). Boxplots for the human and mouse datasets are available from the following link and will be updated with additional datasets: http://dx.doi.org/10.6084/m9.figshare.1521657.

## Supplementary Material

Supplementary information
