# Supplementary material for: Defining the three cell lineages of the human blastocyst by single-cell RNA-seq
Source: Development. 2015 Sep 15;142(18):3151–65. doi: 10.1242/dev.123547 (PMC4582176; doi:10.1242/dev.123547)
Supplement: Supplementary information [file supp_142_18_3151__index.html]

Supplementary information 

# Defining the three cell lineages of the human blastocyst by single-cell RNA-seq

## DEV123547 Supplementary information

- Supplementary information
