# Supplementary material for: Defining the three cell lineages of the human blastocyst by single-cell RNA-seq
Source: Development. 2015 Sep 15;142(18):3151–65. doi: 10.1242/dev.123547 (PMC4582176; doi:10.1242/dev.123547)
Supplement: Supplementary information [file supp_dev.123547_DEV123547supp.pdf]

Single-Cell RNA-seq Defines the Three Cell Lineages of the Human Blastocyst

Paul Blakeley, Norah M.E. Fogarty, Ignacio del Valle, Sissy E. Wamaitha, Tim Xiaoming Hu, Kay Elder, Philip Snell, Leila Christie, Paul Robson and Kathy K. Niakan

Supplementary Materials and Methods

Human embryo culture

Vitrified embryos frozen in straws were thawed by quickly transferring the contents of the straw from liquid nitrogen directly into thaw solution (Irvine Scientific Vitrification Thaw Kit). Embryos frozen in cryopets were first thawed for 3 seconds in a 37°C waterbath and transferred into thaw solution (Irvine Scientific Vitrification Thaw Kit). After 1 min the embryo was transferred from thaw solution into dilution solution for 4 min followed by two washes in wash solution for 4 min each (Irvine Scientific Vitrification Thaw Kit). Embryos frozen in a glass ampoules were thawed completely in a 37°C waterbath after the top of the vial was removed under liquid nitrogen. The contents were emptied onto a petri dish and the embryo transferred through a 0.5 M sucrose solution for 5 minutes, 0.2 M sucrose solution for 10 min and diluent for 10 min (Quinn's Advantage Thaw Kit, Origio). The embryos were cultured in Global Media (LifeGlobal) supplemented with 5 mg/mL LifeGlobal Protein Supplement pre-equilibrated overnight in an incubator at 37ºC and 5% CO2. Embroys cultured in

Development • Supplementary information

Micromanipulation

Single cells were isolated from blastocyst stage embryos (6-7 days post fertilisation) for subsequent analysis by micromanipulation, with a duration of less than 20 minutes. Embryos were placed in drops of G-MOPS solution (Vitrolife) on a petri dish overlaid with mineral oil. The plate was placed on a microscope stage (Olympus IX70) and the embryos were held with an opposing holding pipette and blastomere biopsy pipette (Research Instruments) using Narishige micromanipulators (Narishige, Japan). The biopsy mode of a Saturn 5 laser (Research Instruments) was used to separate the majority of the mural TE from the ICM and polar TE. The ICM and polar TE were washed quickly in PBS without Ca^{2+} and Mg^{2+} (Invitrogen) then placed in 0.05% trypsin/EDTA (Invitrogen) for 5 minutes at room temperature. Trypsin was quenched using Global Media supplemented with 5 mg/mL LifeGlobal Protein Supplement. After quenching, the cell clump was placed back on the stage in a drop of G-MOPS solution and pipetted up and down several times using the blastomere biopsy pipette.

cDNA synthesis and amplification

cDNA was generated from single cells using the SMARTer Ultra Low Input RNA kit for Illumina Sequencing–HV (Clontech Laboratories, Inc.) according to maunfacturers' guidelines. Single cells were picked using 100 µm inner diameter Stripper pipette (Origio) and transferred to individual low bind RNAse-free tube containing 0.25 µl RNase inhibitor, 4.75 µl Dilution buffer and 5 µl nuclease-free water on a -80°C pre-chilled CoolRack (Biocision, CA). Samples were stored at -80°C until ready to be processed. 1 µl of 3' SMART CDS Primer II A was added to

Development • Supplementary information

the sample, mixed well and incubated at 72°C for 3 min. First strand cDNA was synthesised by adding 4 µl 5X First-Strand Buffer, 0.5 µl 100 mM DTT, 1 µl 20 mM dNTP mix, 1 µl SMARTer IIA Oligonucleotide, 0.5 µl RNase Inhibitor and 2 µl SMARTScribe Reverse Transcriptase (100 U/µl) directly to a tube containing the sample and incubating at 42°C for 2 hours followed by 10 min at 70°C.

First strand cDNA was purified by adding 36 µl of room temperature SPRI Ampure XP beads (Beckman Coulter Genomics), mixing well and incubating at room temperature for 8 min. Tubes were placed on a MagnaBot II Magnetic Separation device (Promega) and allowed to stand until all beads were immobilised into a pellet. The supernatant was removed and discarded. Tubes were briefly spun and any residual liquid was removed.

Double stranded cDNA was amplified from the template bound to the beads using Advantage 2 PCR kit (Clontech Laboratories, Inc.). 5 µl 10X Advantage 2 PCR Buffer, 2 µl 10 mM dNTP Mix, 2 µl IS PCR Primer, 2 µl 50X Advantage 2 Polymerase Mix and 39 µl Nuclease-Free water were added to the tube containing the sample to give a total volume of 50 µl. PCR amplification was performed at 95°C for 1 min, followed by 18 cycles of 15 sec at 95°C, 30 sec at 65°C and 6 min at 68°C followed by final extension step of 10 min at 72°C. Amplified cDNA was purified by adding 90 µl SPRI Ampure XP beads, mixing well and incubating at room temperature for 8 min to allow amplified cDNA bind to the beads. Sample tubes were placed on the magnet and allowed to stand until all beads had been immobilised. Supernatant was removed and discarded and beads were washed twice by adding 200 µl freshly prepared 80% ethanol and leaving for 30 sec before discarding the

supernatant. Tubes were spun briefly to collect residual liquid. The bead pellet was allowed to air dry. 12 µl of purification buffer was added to rehydrate the pellet and incubated for 2 min at room temperature. cDNA was eluted by pipetting up and down 10 times before returning the tube to the magnet. The clear supernatant containing the cDNA was removed from the immobilised beads and transferred to a new low-bind tube. cDNA was stored at -80°C until library preparation.

cDNA quality was assessed by High Sensitivity DNA assay on an Agilent 2100 Bioanalyser with good quality cDNA showing a broad peak from 300 to 9000 bp. cDNA concentration was measured using QuBit dsDNA HS kit (Life Technologies UK Ltd.) Typical yields from a single cell ranged from 1 ng to 9 ng.

cDNA shearing and library preparation

In preparation for library generation cDNA was sheared using Covaris S2 to achieve cDNA in 200-500 bp range. 10 µl of cDNA sample and 65 µl purification buffer was added to Covaris AFA Fiber Pre-Slit Snap Cap microTUBE. cDNA was sheared using the settings 10% Duty, Intensity 5, Burst Cycle 200 for 2 min. Sheared cDNA was transferred to a new 0.2 ml low-bind tube.

Libraries were prepared using Low Input Library Prep Kit (Clontech Laboratories, Inc.) according to manufacturer's instructions. The amount of input cDNA was calculated from the concentration measured by the Bioanalyser assay prior to shearing, taking into account the dilution involved in the shearing step. The appropriate amplification cycle number was selected according to manufacturer's

guidelines. Library quality was assessed by Bioanalyser and the concentration was measured by QuBit assay. The molar concentration of library was calculated thus:

Library molecular weight = average size in bp (from Bioanalyser) x 650 g/mol per bp

Molar concentration = library concentration from QuBit/library molecular weight

Libraries with a molar concentration greater than 2nM were submitted for 50-bp paired-end sequencing on Illumina HiSeq 2000.

Data acquisition and processing

We integrated previously published datasets with our own blastocyst sequencing data using a consistent read alignment method. SRA files were obtained via ftp from the Gene Expression Omnibus, under the accession numbers GSE36552 and GSE45719. The SRA files were converted into FASTQ format using the fastq-dump program from the SRA toolkit (http://www.ncbi.nlm.nih.gov/Traces/sra/). The reference human genome sequence was obtained from Ensembl, along with the gene annotation (GTF) file. The reference sequence was indexed using the bowtie2-build command.

Read mapping and counting

Reads were aligned to the reference human genome sequence using Tophat2 (Kim et al., 2013), with gene annotations to obtain BAM files for each of the single-cell samples. BAM files were then sorted by read coordinates and converted into SAM files using SAMtools. The process of mapping and processing BAM files was automated using a custom Perl script. The number of reads mapping to each gene were counted using the program htseq-count (Anders et al., 2015). The resulting count files for each sample were used as input for differential expression analysis using DESeq using the hg19 human or mm9 mouse genome reference sequence.

Development • Supplementary information

Expression analysis

To investigate differences in global gene expression, a PCA of the top 8000 genes with the most variable expression was performed on the human and mouse RPKM data separately. The R package prcomp was used to generate the PCA, using both the scaling and centering options. The R package NOISeq (Tarazona et al., 2011) was used to identify genes differentially expressed between the TE and EPI cells in human, and between TE and ICM cells in mouse. To increase sensitivity, genes with an RPKM > 5 in four or more samples were retained for NOISeq analysis. Differentially expressed genes were identified after applying a 95% probability threshold. Ensembl Biomart was used to find human-mouse orthologous pairs within the list of differentially expressed human and mouse gene.

We used a second independent method to detect differentially expressed genes between human EPI and TE using DESeq (Anders and Huber, 2010). Firstly, the function 'estimateSizeFactors' and 'estimateDispersions' were used to estimate biological variability and calculate normalised relative expression values across the different blastocyst samples. Initially, this was performed without sample labels (option: *method='blind'*) to allow unsupervised clustering of the blastocyst samples using principal components analysis and hierarchical clustering. The dispersion estimates were recalculated with the sample labels included and with the option: *method='pooled'*. The function 'nbinomTest' was then used to calculate p-values to identify genes that show significant differences in expression between different cell types.

Development • Supplementary information

A k-means clustering analysis was performed to find clusters of genes co-expressed during pre-implantation development. The mean RPKM value for each developmental time point was calculated for subsequent k-means clustering analysis (Figs S1, S2). Genes with a fold change of greater than two between any two stages were retained. The R package 'MFuzz' was used to generate the k-means clusters using the kmeans2 function, with the number of clusters set to 50. A custom R script was used to generate plots for the k-means clusters and trendlines were drawn based on the k-means centroids. The k-means clusters were clustered further using the R function 'hclust' and heatmaps were generated using R package 'pheatmap'.

PCA comparison of EPI versus hESC gene expression

We compared the EPI single cell RNA-seq dataset to distinct hESC lines (Yan et al., Takashima et al. and Chan et al.) (GSE36552, E-MTAB-2857, E-MTAB-2031). These data were processed using our computational pipeline to generate read counts and RPKM values for each gene. NOISeq was used to perform differential expression analysis of the EPI versus hESC samples. Samples from Yan et al. were grouped into early or late hESCs, and NOISeq analysis was performed independently on these two groups. Samples from Chan et al. and Takashima et al. were grouped into primed or reset hESCs, and NOISeq analysis was performed independently on each of the groups. RPKM values for genes showing differential expression in least one of pairwise test were used to generate a PCA plot, showing the relationship of gene expression between the hESC lines and the EPI. In addition, the Pearson correlation coefficient was calculated using the median RPKM between each pair of conditions. The R function 'pairs' was used to generate scatterplots comparing the median RPKMs between each pair of conditions.

Development • Supplementary information

Pathway enrichment analysis

The GSEA method (Subramanian et al. 2005) was used to identify pathways and GO terms enriched in each hESC line versus the human EPI, the human trophectoderm versus the human EPI or the mouse ICM versus the TE. We used a compendium of multiple pathway interaction databases downloaded from the Bader Lab: http://baderlab.org

Genes were ranked according to log2fold change between the each pairwise comparison. The resulting .rnk files were then used as input for the GSEAPreranked module and enrichment analysis was performed. The GSEA output files were loaded into the Cytoscape module EnrichmentMap (Shannon, P. 2003; Merico, D. 2010) and the relationship between the expressed signaling pathways was displayed in an interaction network map.

DESeq Analysis

For DESeq analysis, firstly, the function ‘estimateSizeFactors’ and ‘estimateDispersions’ were used to estimate biological variability and calculate normalised relative expression values across the different blastocyst samples. Initially, this was performed without sample labels (option: *method=’blind’*) to allow unsupervised clustering of the blastocyst samples using principal components analysis and hierarchical clustering. The dispersion estimates were recalculated with the sample labels included and with the option: *method=’ pooled’*. The function ‘nbinomTest’ was then used to calculate p-values to identify genes, which show significant differences in expression between different cell types.

Development • Supplementary information

Quantification of Immunofluorescence

MINS 1.3 software was used to detect and segment nuclei and generate tables of fluorescence intensity for each channel (http://katlab-tools.org/) (Lou et al Stem Cell Reports 2014). Embryos were imaged at a z-section thickness of 3µm. Confocal stacks in .tif format were loaded into the MINS pipeline for automated nuclear segmentation. The MINS segmentation output was manually checked for appropriate segmentation and tables were amended accordingly. Mitotic nuclei were removed from the analysis and the background adjusted using a method described previously (Schrode et al., Dev Cell 2014). Data were subsequently plotted using GraphPad Prism version 6 (GraphPad Software, La Jolla, CA).

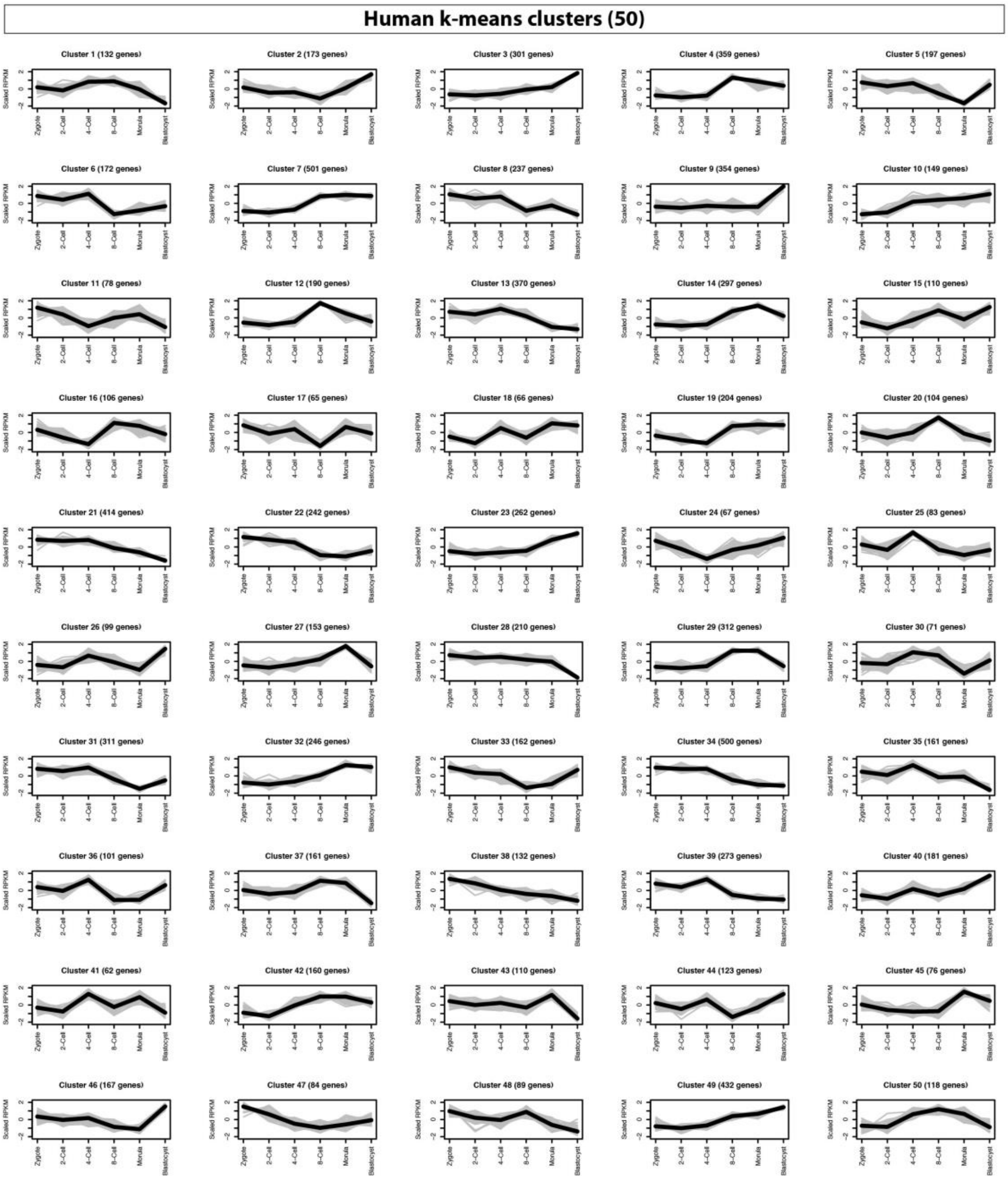

Figure S1. Time course plots for 50 k-means clusters generated from the human single-cell RNA-seq dataset. RPKM values were averaged for each developmental stage and invariable genes filtered prior to clustering. Black lines indicate median expression profiles.

Development • Supplementary information

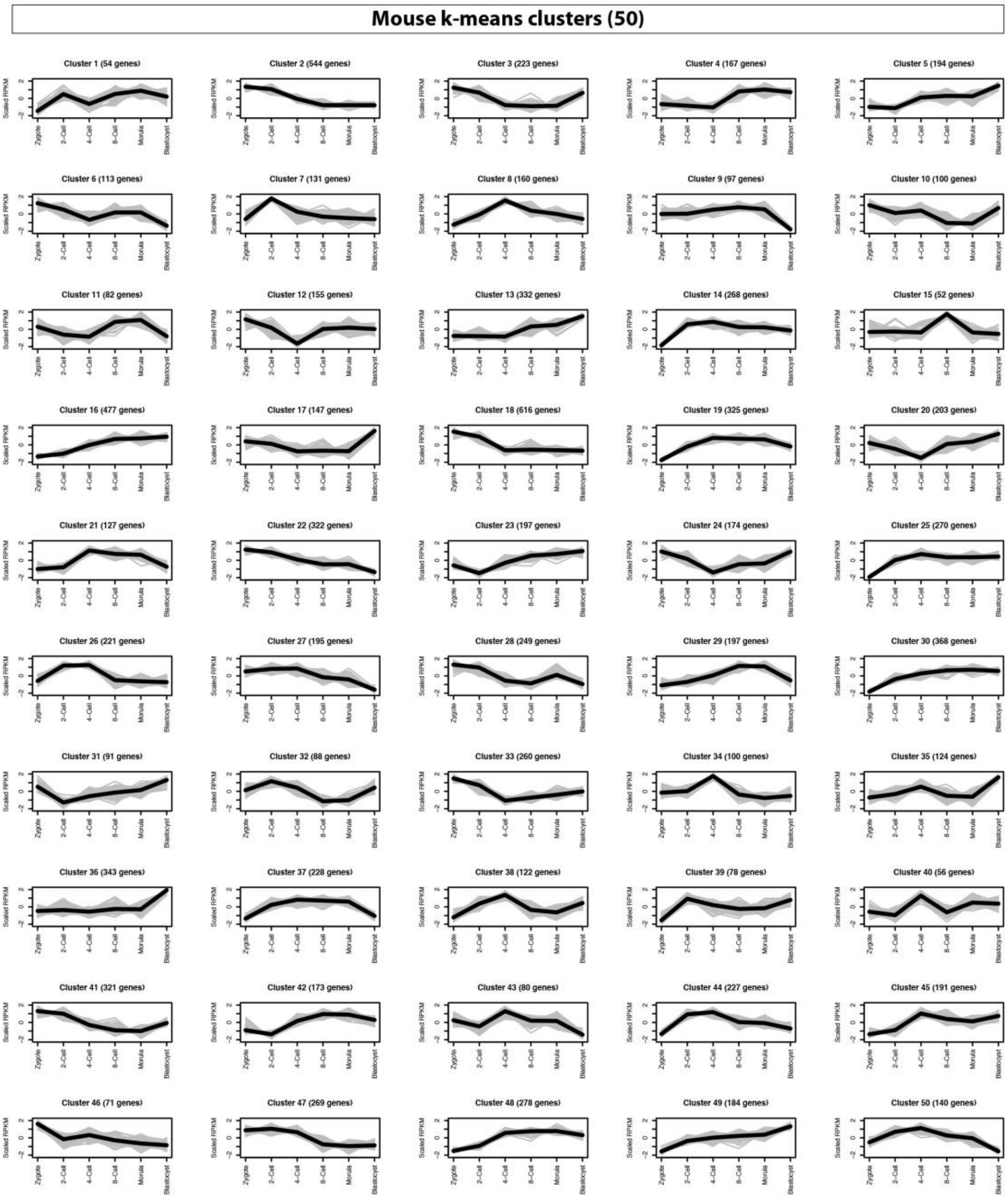

Figure S2. Time course plots for 50 k-means clusters generated from the mouse single-cell RNA-seq dataset. RPKM values were averaged for each developmental stage and invariable genes filtered prior to clustering. Black lines indicate median expression profiles.

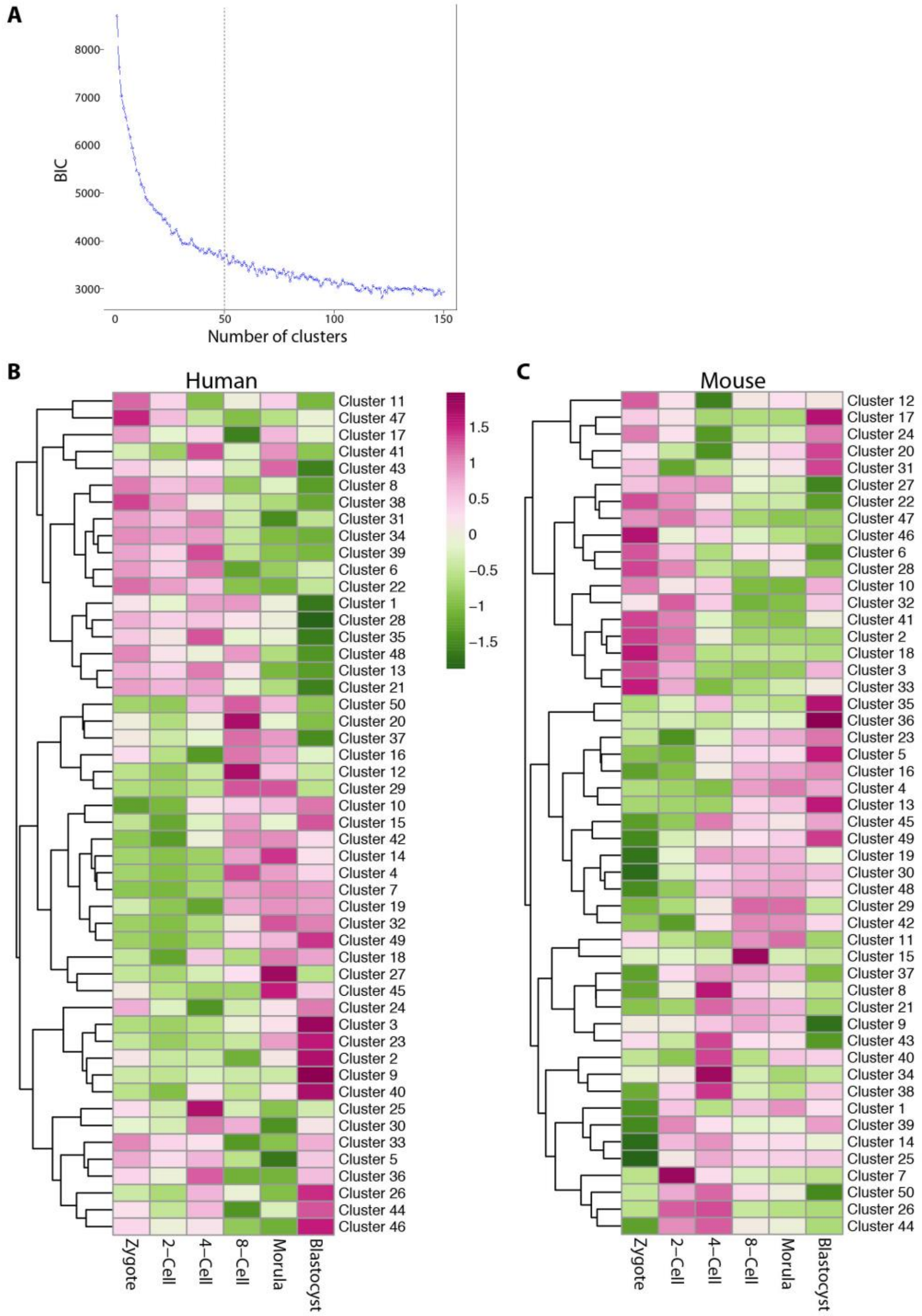

Figure S3. (A) Estimating the optimum number of k-means clusters based on the Bayesian Information Criterion (BIC). RPKM values from from the human dataset were averaged within each developmental stage and invariable genes filtered prior to BIC estimation. The BIC score was plotted for up to 150 clusters and shows the inflection point lies at approximately 40-50 clusters. (B-C) Heatmap of the hierarchical clustering of (B) human and (C) mouse k-means clusters reveals global gene expression patterns across time. Expression levels were plotted on a high-to-low scale (purple-white-green).

Development • Supplementary information

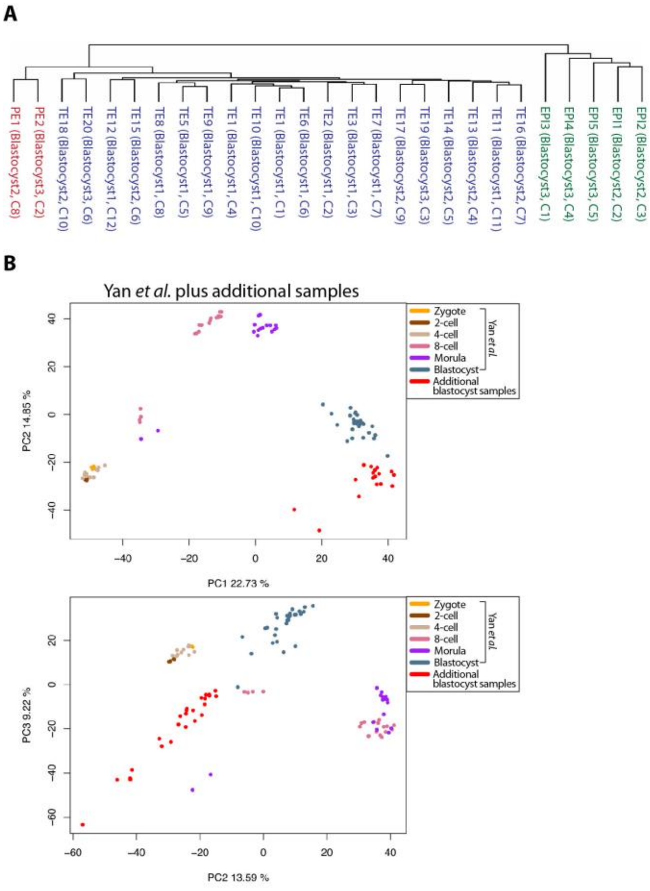

C

	Gene ID	Epi Mean	TE Mean	Log2 FC	p-value
EPI enriched	*NANOG*	6741.3	9.7	9.4	2.7E-49
	ETV4	741.9	27.4	7.1	3.1E-2
	KLF17	2043.7	9.2	7.8	1.9E-10
	PRDM14	1817.8	6.7	8.1	3.0E-06
	FOXD3	574.9	0.2	11.2	6.8E-06
	POU5F1	3499.7	427.8	3.0	2.7E-05
	SOX2	1100.3	11.1	6.6	3.2E-05
TE enriched	*GATA2*	16.1	4528.6	8.1	2.2E-19
	GATA3	255.1	8457.3	5.1	4.1E-13
	LRIG3	5.1	927.4	7.5	1.9E-07
	TEAD1	177.9	2934.3	4.0	2.7E-05
	CDX2	2.5	431.9	7.4	4.7E-04
	KRT18	3463.5	46649.9	3.8	8.6E-07
	CLDN4	7.9	611.5	6.3	1.5E-06

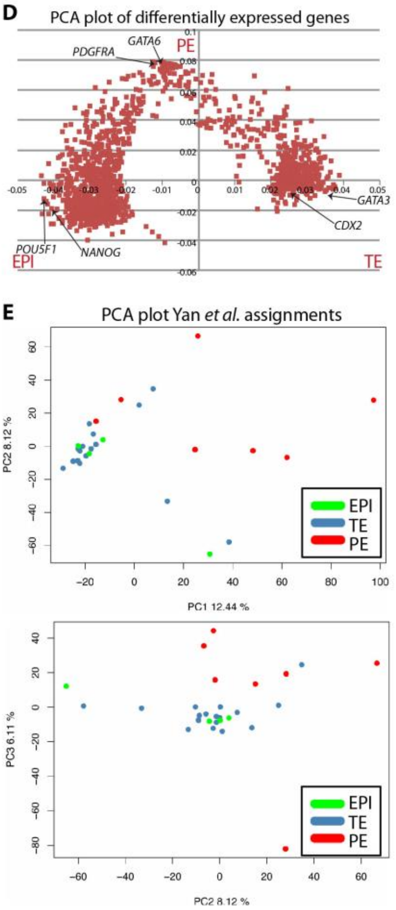

Figure S4. Lineage-specific gene expression from the single-cell RNA-seq data. (A) Dendrogram representing hierarchical clustering of human blastocyst single-cell samples from Yan *et al.*, 2013. (B) PCA incorporating our additional single-cell RNA-seq samples (red) together with the samples (zygote to blastocyst) from Yan *et al.*, 2013. (C) Differentially expressed genes identified by DESeq. The log2 fold change (FC) difference in expression is noted. (D) Loading scores for first two principal components based on the human RNA-seq data. Each point represents the loading score for a single gene. (E) PCA of single-cell RNA-seq data from blastocyst samples, coloured according to lineage assignments made by Yan *et al.*, 2013.

Development • Supplementary information

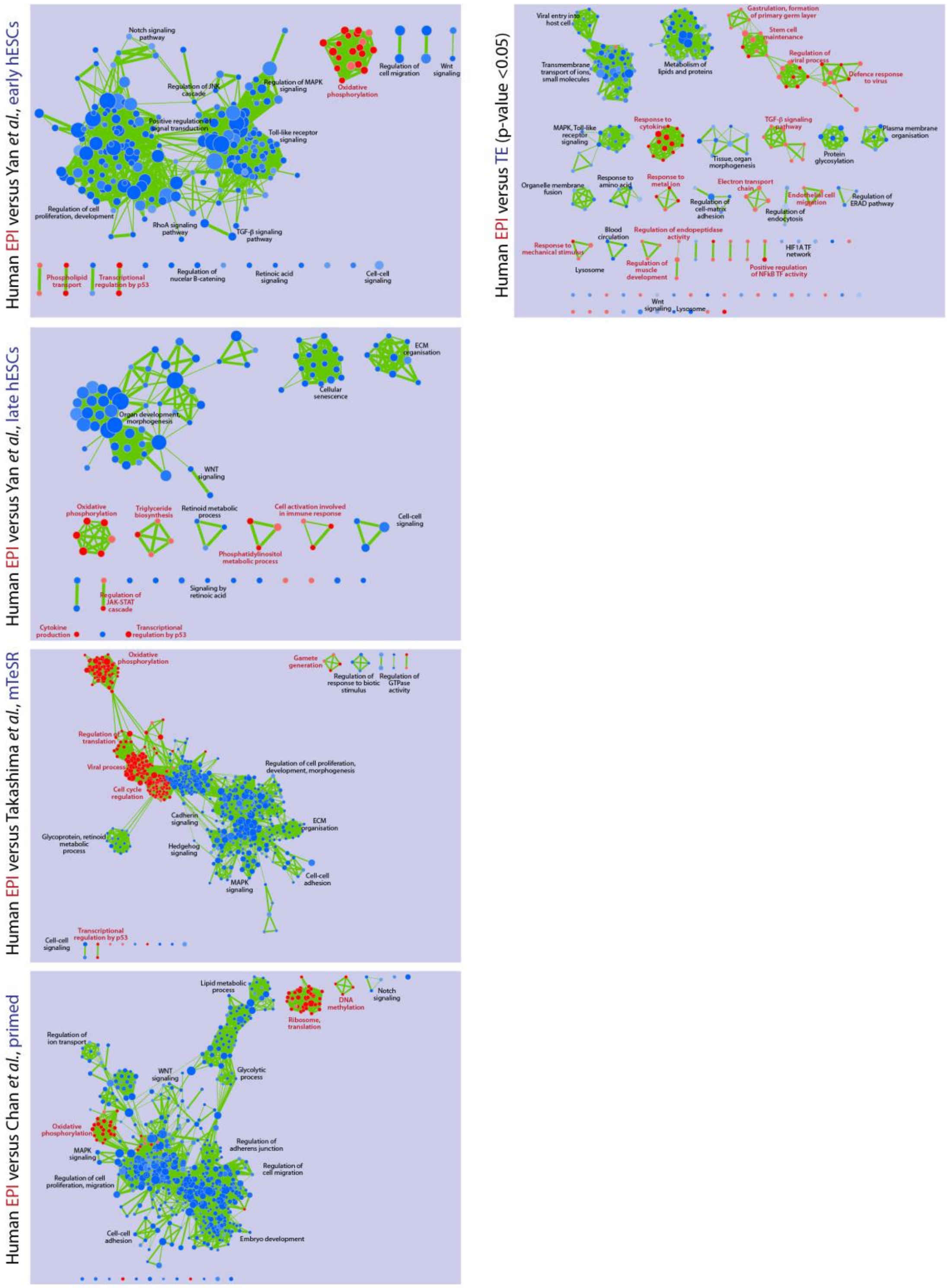

Figure S5. Cytoscape enrichment map of GSEA results comparing epiblast (EPI, red) versus human embryonic stem cells (blue) (p-value < 0.01) or EPI versus TE (p-value < 0.05). Differentially expressed genes were identified using gene set enrichment analysis using a combination of pathways compiled by the Bader lab (http://baderlab.org).

Development • Supplementary information

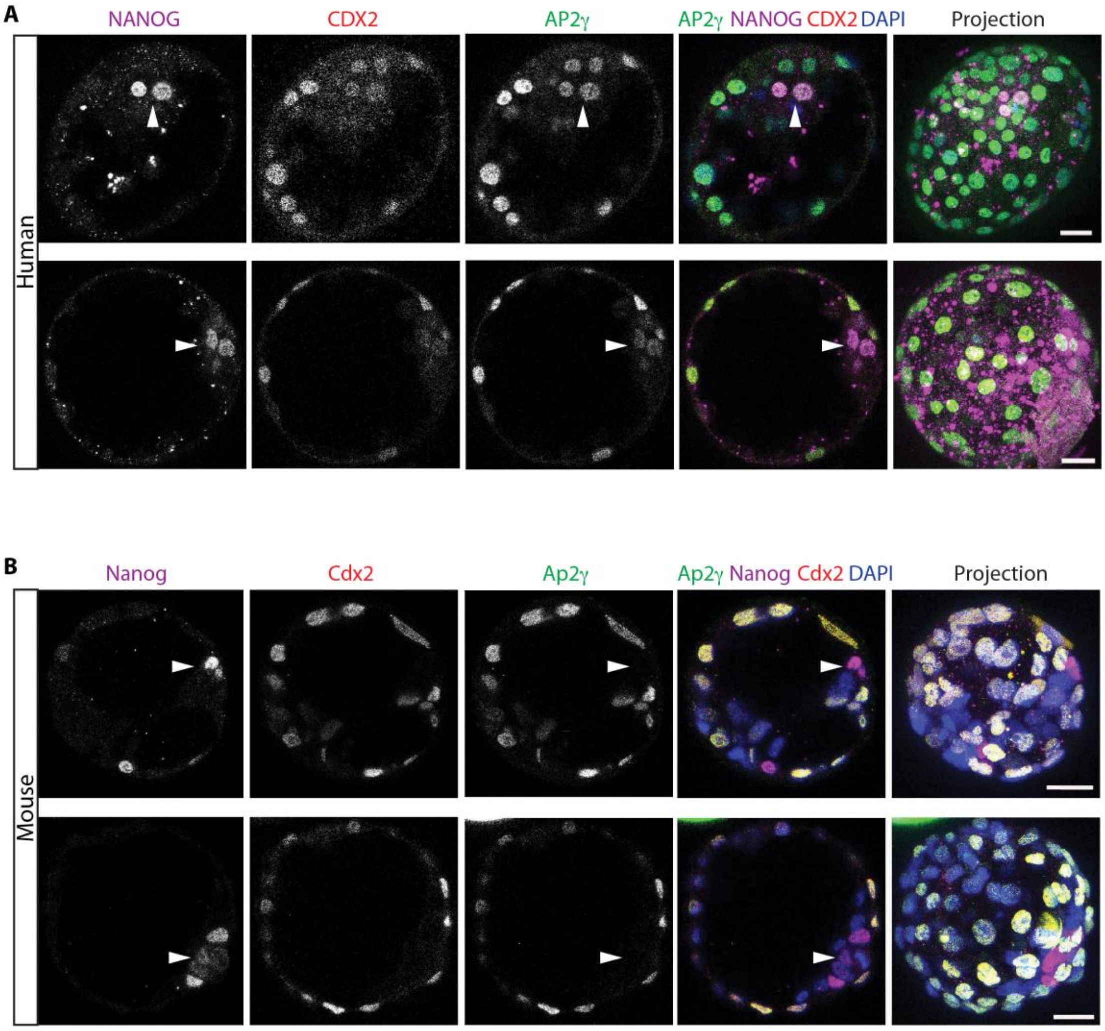

Figure S6. Immunofluorescence analysis of (A) human and (B) mouse blastocysts. The expression of Ap2γ/AP2γ, Nanog/NANOG, Cdx2/CDX2 or DAPI are indicated in green, purple, red or blue, respectively. The merged image and projection of expression is shown. The scale bar is: 25 μm.

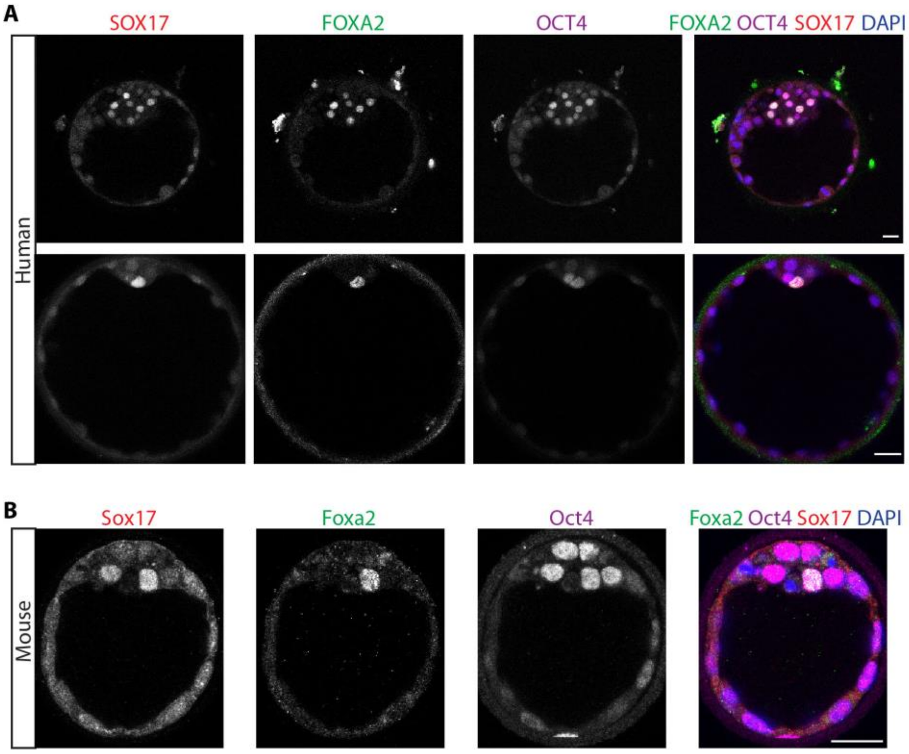

Figure S7. Immunofluorescence analysis of (A) human and (B) mouse blastocysts. The expression of Foxa2/FOXA2 (green), Oct4/OCT4 (purple), Sox17/SOX17 (red) or DAPI (blue) with merged images. Scale bar: 25 µm.

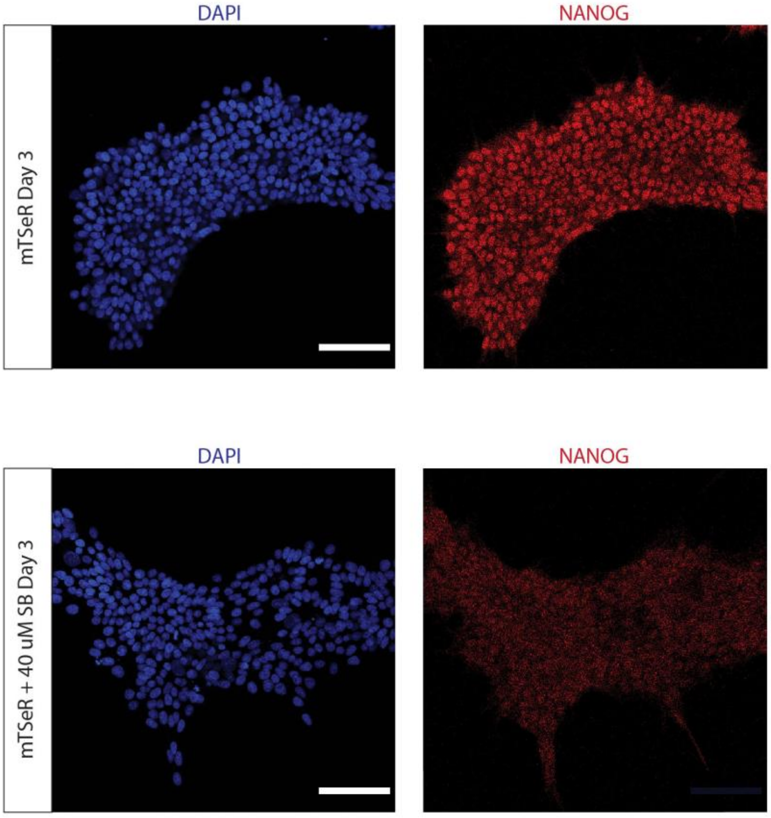

Figure S8. Human embryonic stem cells (H9) were cultured on Matrigel coated glass bottom microwell dishes (MatTek) in mTeSR for 3 days in the presence or absence of 40 μM SB-431542 then immunofluorescently analysed for the expression of NANOG (red) and DAPI (blue). Scale bar: 100 μm.

Table S1

Click here to Download Table S1

Table S2

Click here to Download Table S2

Table S3

Click here to Download Table S3

Table S4

Click here to Download Table S4

Table S5

Click here to Download Table S5

Development • Supplementary information
